# The Synthesis and Biological Applications of the 1,2,3-Dithiazole Scaffold

**DOI:** 10.3390/molecules28073193

**Published:** 2023-04-03

**Authors:** Andreas S. Kalogirou, Hans J. Oh, Christopher R. M. Asquith

**Affiliations:** 1 Department of Life Sciences, School of Sciences, European University Cyprus, 6 Diogenis Str., Engomi, P.O. Box 22006, Nicosia 1516, Cyprus; a.kalogirou@euc.ac.cy; 2 Department of Pharmacology, School of Medicine, University of North Carolina, Chapel Hill, NC 27599, USA; hjo0729@live.unc.edu; 3 School of Pharmacy, Faculty of Health Sciences, University of Eastern Finland, 70211 Kuopio, Finland

**Keywords:** antibacterial, anticancer, antifibrotic, antifungal, antimicrobial, antiviral, appel salt, 1,2,3-dithiazole, disulfide bridge, herbicidal

## Abstract

The 1,2,3-dithiazole is an underappreciated scaffold in medicinal chemistry despite possessing a wide variety of nascent pharmacological activities. The scaffold has a potential wealth of opportunities within these activities and further afield. The 1,2,3-dithiazole scaffold has already been reported as an antifungal, herbicide, antibacterial, anticancer agent, antiviral, antifibrotic, and is a melanin and Arabidopsis gibberellin 2-oxidase inhibitor. These structure activity relationships are discussed in detail, along with insights and future directions. The review also highlights selected synthetic strategies developed towards the 1,2,3-dithiazole scaffold, how these are integrated to accessibility of chemical space, and to the prism of current and future biological activities.

## 1. Introduction

The 1,2,3-dithiazole core is a five membered heterocycle containing two sulfur atoms and one nitrogen atom. Despite the fact that the 1,2,3-dithiazole is not present in nature, similar to many other heterocycles, it does have a broad range of interesting biological activities. The 1,2,3-dithiazole moiety was first synthesized in 1957 by G. Schindler et al. [1]. This was followed two decades later by a report by J. E. Moore on behalf of Chevron Research Co. (San Ramon, CA, USA) where it showcased antifungal and herbicidal activity [2,3]. In 1985, Appel et al. reported the synthesis of 4,5-dichloro-1,2,3-dithiazolium chloride **1** (Appel’s salt), a precursor which allowed access to the 1,2,3-dithiazole core within a single step [4,5].

**Figure 1:**
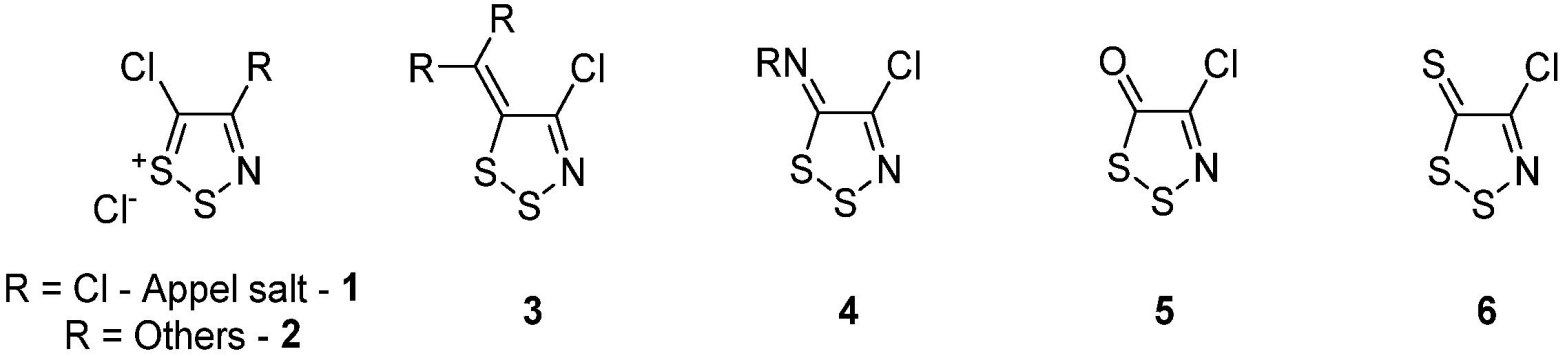
Appel salt (**1**) and other general 1,2,3-dithazoles structures **2**–**6**.

The synthesis of Appel salt **1** acted as a catalyst to the field and granted access to many 1,2,3-dithiazole derivatives, and to other heterocycles incorporating sulfur and nitrogen atoms [6,7,8,9,10,11]. The subsequent synthetic reports focused on transformations on the C5 position [6,7,8,9,10,11]. However, one of the key synthetic interests beyond expanding the scope of 5-substituted 1,2,3-dithiazoles was the limited reactivity of the C4 position. Several different approaches were used to address this C4 reactively issue, including intramolecular cyclization [6] using a multi-step oxime pathway [12,13], or more recently, direct reactions [14], all of which expanded the chemical space around the 1,2,3-dithiazole. Some of these approaches have been covered in past reviews around the chemistry of 1,2,3-dithiazoles [6,7,8,9,10,11] (Figure 1).

Despite the remaining synthetic challenges, the 1,2,3-dithiazole scaffold has already been reported as an antifungal [2], herbicide [2], antibacterial [15], anticancer agent [16], antiviral [17], antifibrotic [18], and as a melanin [19] and Arabidopsis gibberellin 2-oxidase [20] inhibitor. While there is a wide range of existing biology, there are a wealth of opportunities for expansion, including broader application toward cystine reactive sites [21,22,23,24,25]. In this review, we are primarily focused on the impact of: (1) The chemistry limiting the chemical space, and hence, limiting the biology; (2) The chemistry impacting the biology observed; and (3) How chemistry could be applied to new biology. The chemistry, biology, structure activity relationships, and future directions of research in 1,2,3-dithiazoles are all outlined below.

## 2. 1,2,3-Dithiazoles Synthesis Overview

### 2.1. Early Years before Appel Salt

**Scheme 1:**
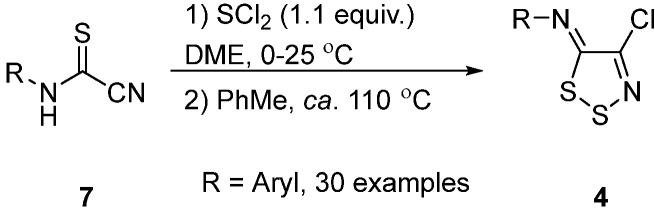
Synthesis of *N*-aryl-5*H*-1,2,3-dithiazol-5-imines **4** from arylcyanothioformamides **7**.

Early work on the synthesis of 1,2,3-dithiazoles used cyanothioformamides as starting materials. Treatment of a variety of arylcyanothioformamides **7** with sulfur dichloride at 0–25 °C gave a number of *N*-aryl-5*H*-1,2,3-dithiazol-5-imines **4** (Scheme 1) [2]. The initial reaction yielded the corresponding hydrochloride salts, which could be converted to the free base by refluxing in a toluene solution.

**Scheme 2:**
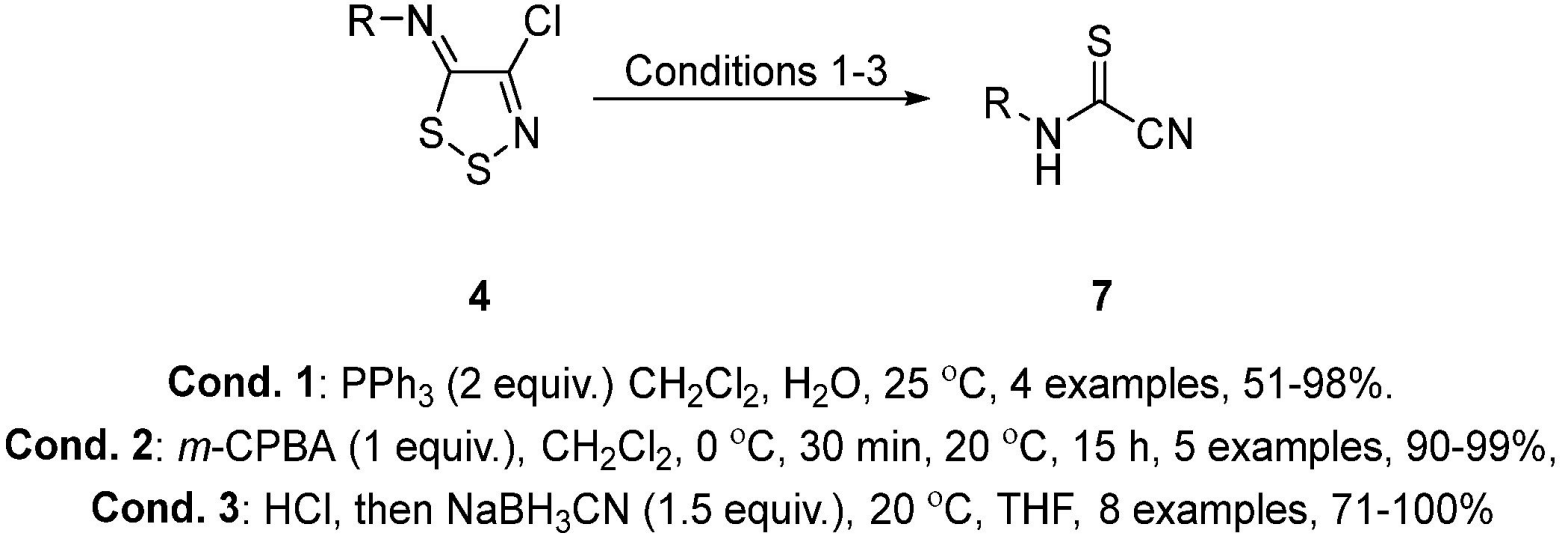
Degradation of *N*-aryl-5*H*-1,2,3-dithiazol-5-imines **4** to cyanothioformamides **7**.

Interestingly, the *N*-aryl-5*H*-1,2,3-dithiazol-5-imines **4** can be degraded to the respective cyanothioformamides **7** by thiophilic ring cleavage after reaction with triphenylphosphine or sodium hydroxide [4,26], oxidative ring cleavage after reaction with *m*-CPBA [27], or by reductive ring cleavage after reaction with sodium cyanoborohydride [28] (Scheme 2).

### 2.2. Discovery of Appel Salt and Applications

**Scheme 3:**
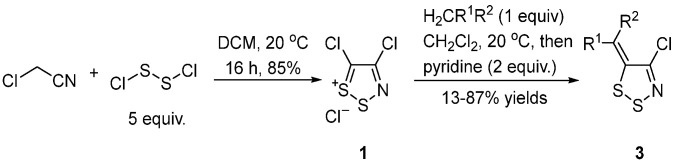
Synthesis of Appel salt and transformation to 4-chloro-5*H*-1,2,3-dithiazolylidenes **3**.

A significant discovery in the chemistry of 1,2,3-dithiazoles was the synthesis of 4,5-dichloro-1,2,3-dithiazolium chloride **1** (Appel salt) by Appel et al. in 1985, which was readily prepared from chloroacetonitrile and disulfur dichloride [4,29] (Scheme 3). Appel salt **1** was subsequently used as an important reagent for the preparation of other 4-chloro-5*H*-1,2,3-dithiazoles with the most reactive site being the electrophilic C-5 position [4,9,27].

Appel salt **1** can condense with active methylenes, such as acetonitrile derivatives [4,30,31], diketones, ketoesters, and others [32], to give 4-chloro-5*H*-1,2,3-dithiazolylidenes **3** (Scheme 3).

**Scheme 4:**
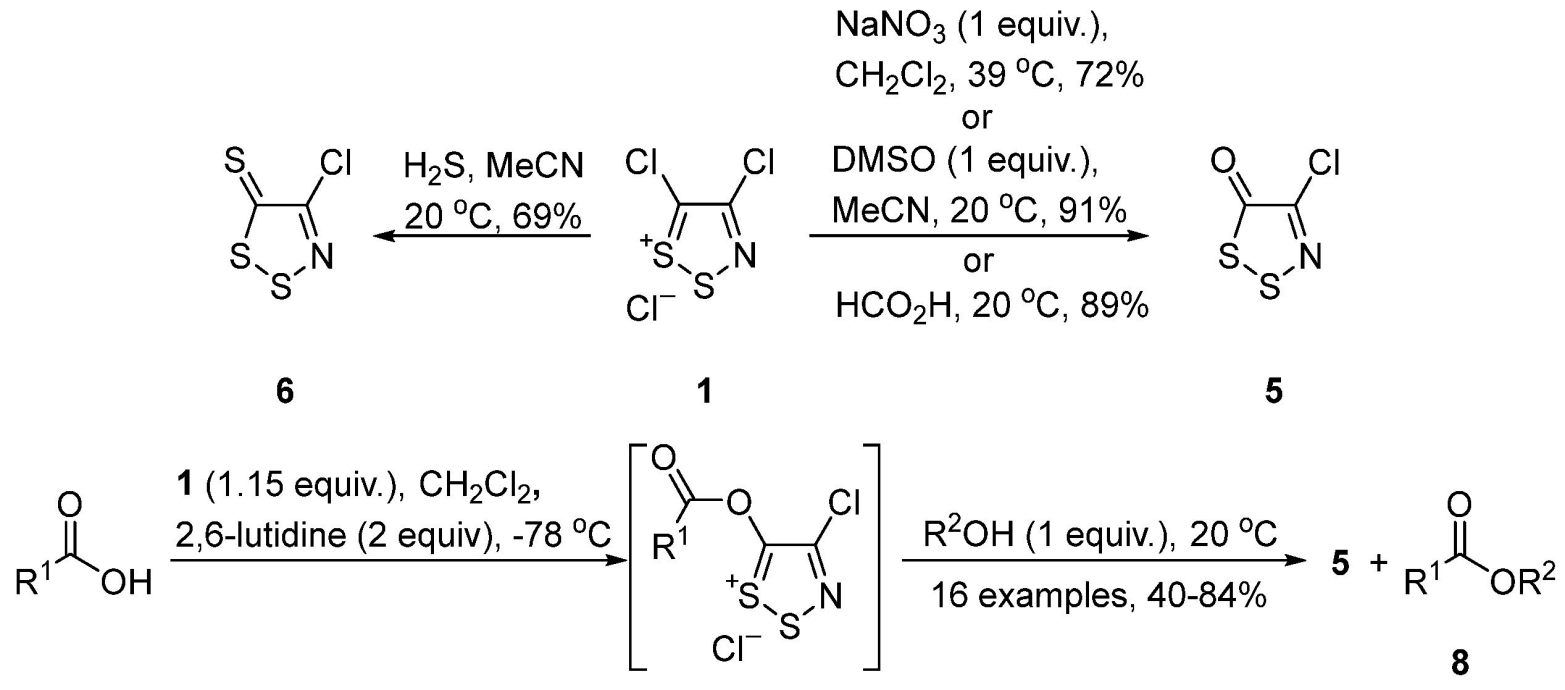
Reactions of Appel salt **1** with oxygen and sulfur nucleophiles.

The condensation of Appel salt with hydrogen sulfide [4] afforded dithiazole-5-thione **6** in 69% yield (Scheme 4). The reaction with oxygen nucleophiles are also common with NaNO_3_ [4], sulfoxides [33], or formic acid [34] all acting as the source of oxygen to give 4-chloro-5*H*-1,2,3-dithiazol-5-one **5** in good yields (Scheme 4). Furthermore, the reaction with other carboxylic acids [35] at −78 °C and subsequent treatment with alcohols gave esters **8** in medium to good yields (Scheme 4).

**Scheme 5:**
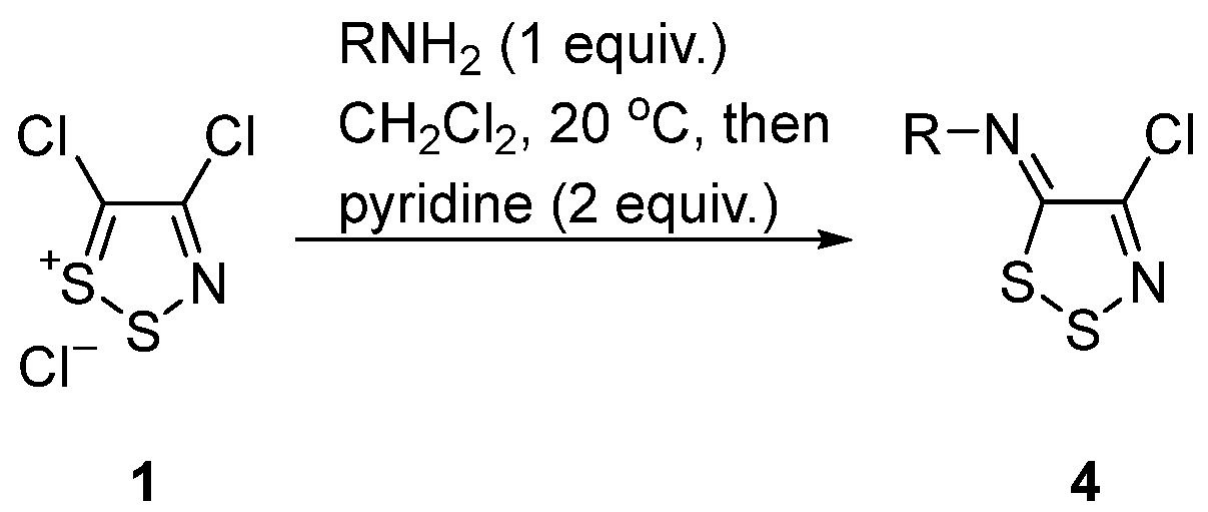
Synthesis of *N*-aryl-5*H*-1,2,3-dithiazol-5-imines **4** from Appel salt **1**.

The condensation of Appel salt **1** with primary anilines is well studied [4,5,15,36] and typically occurs by treatment with 1 equiv. of the aniline in the presence of pyridine (2 equiv.) as the base to give, in most cases, good yields of *N*-aryl-5*H*-1,2,3-dithiazol-5-imines **4** (Scheme 5).

**Scheme 6:**
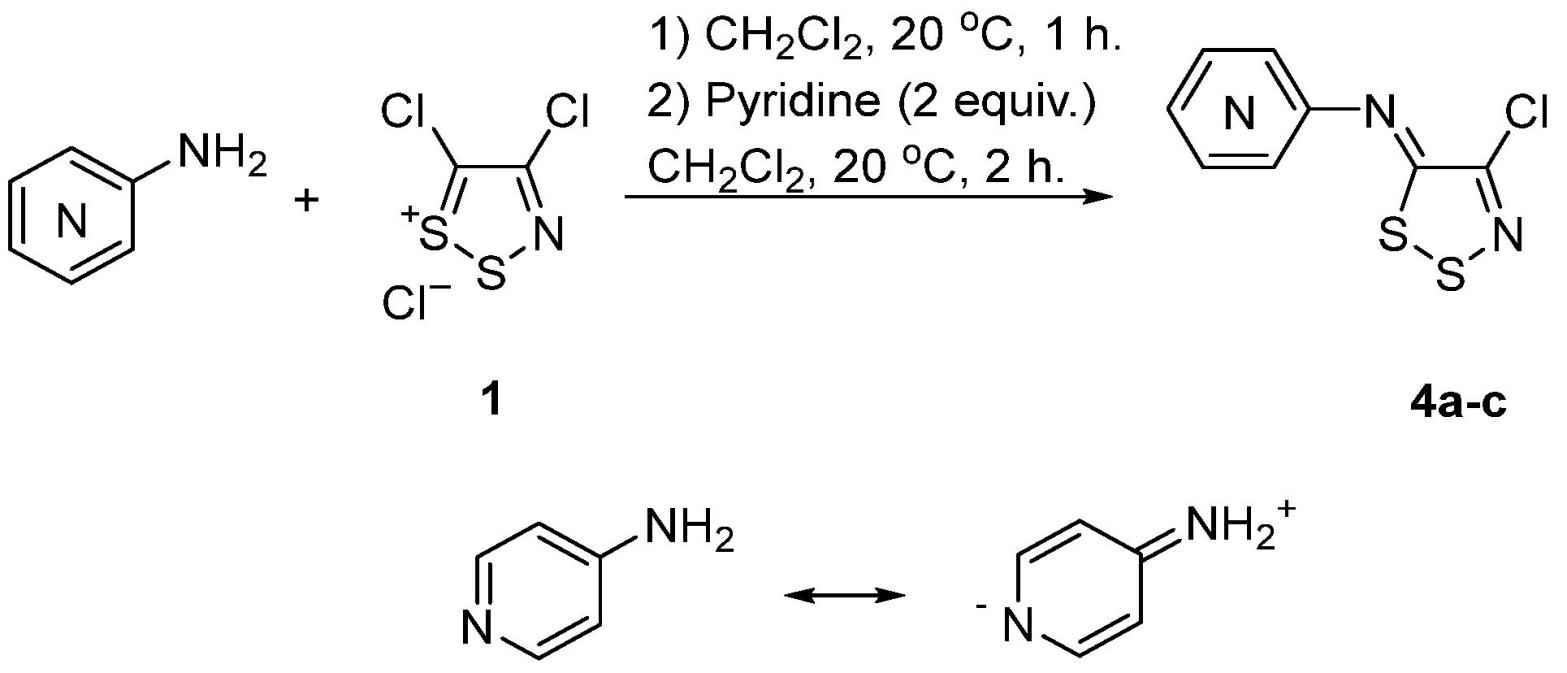
Reaction of Appel salt **1** with aminopyridines.

Some limitations of this chemistry appear when using heterocyclic arylamines, such as aminopyridines. A recent study by Koutentis et al. highlighted that the reactions of the three isomeric aminopyridines with Appel salt **1** gave very different yields based on the position of the amino group. The 2-, 3- and 4-aminopyridines gave 69%, 24%, and 1% yields of the desired 1,2,3-dithiazole, respectively [37] (Scheme 6). Koutentis et al. suggested the low yield of 4-aminopyridine is likely attributed to the reduced nucleophilicity of the primary amine due to a contribution of its zwitterionic resonance form. The low reactivity of the amine leads to complex reaction mixtures due to side reactions.

### 2.3. Reactivity of C-4 and the Displacement of the Chloride

**Scheme 7:**
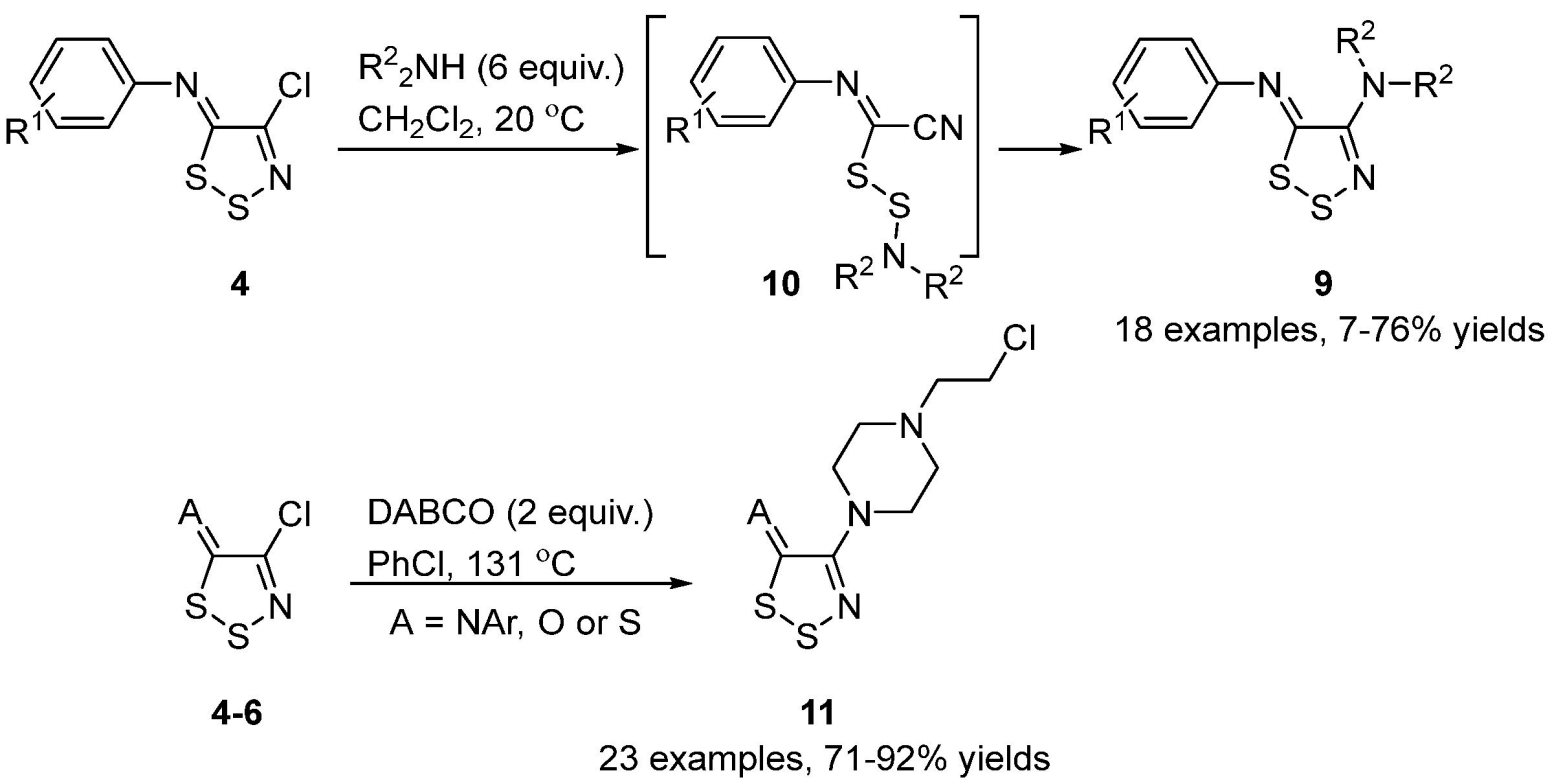
Displacement of the C4 chlorine of neutral 5*H*-1,2,3-dithiazoles.

The less reactive C4 chlorine of neutral 5*H*-1,2,3-dithiazoles cannot be directly substituted by nucleophiles. However, utilizing an ANRORC-(Addition of the Nucleophile, Ring Opening, and Ring Closure)-style mechanism, nucleophilic substitution can occur on the C4 chlorine of the 1,2,3-dithazole. An example of this is where the *N*-Aryl-5*H*-1,2,3-dithiazol-5-imines **4** react with an excess of dialkylamines to give 4-aminodithiazoles **9** in variable yields (Scheme 7). The reaction was found to proceed *via* an ANRORC-style mechanism [38,39] involving ring opening by nucleophilic attack on the S2 position to yield disulfides **10** and subsequent recyclization after amine addition on the cyano group [40]. In another report by Koutentis et al. [14], DABCO was reacted with neutral 5*H*-1,2,3-dithiazoles **4**–**6** to give *N*-(2-chloroethyl)piperazines **11** in good yields (Scheme 7). The chloroethyl group originating from chloride attack on the intermediate quaternary ammonium salt formed by the displacement of the C4 chloride by DABCO.

### 2.4. Alternatives beyond Appel Salt Chemistry

**Scheme 8:**
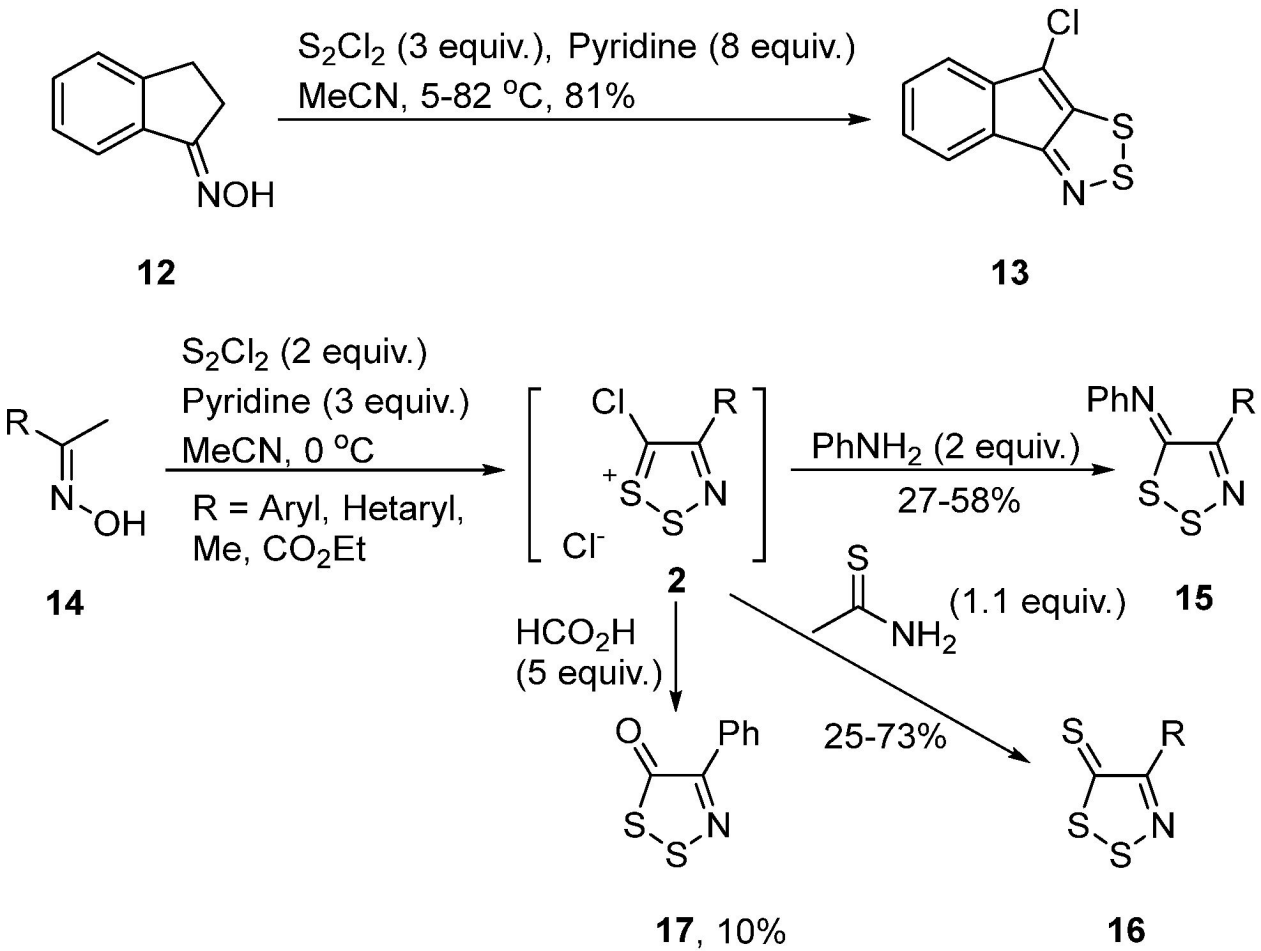
Synthesis of 1,2,3-dithiazoles from oximes.

A different way to access both monocyclic and ring fused 1,2,3-dithiazoles is by the reaction of oximes with disulfur dichloride. An example of the synthesis of a ring fused dithiazole is the reaction of benzoindenone oxime **12** to give dithiazole **13** in 81% yield [41,42] (Scheme 8). Acetophenone oximes **14** were reacted with disulfur dichloride to yield dithiazolium chlorides **2**, which were subsequently converted to either imines **15**, thiones **16**, or ketone **17** [13] (Scheme 8). Insights in the mechanism of the oxime to dithiazole transformation were given by Hafner et al. [12], who isolated the dithiazole *N*-oxide, which is the intermediate in this reaction.

### 2.5. Reactivity of 1,2,3-Dithiazoles

**Scheme 9:**
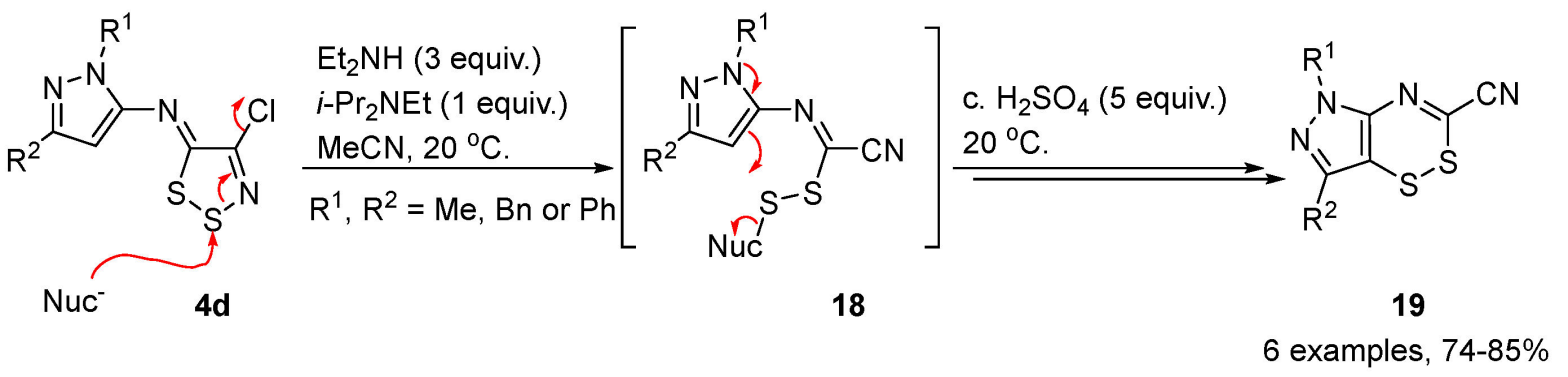
Synthesis of 1,2,4-dithiazines **19**.

Neutral 1,2,3-dithiazoles can also be transformed to a plethora of other heterocycles, often substituted by a cyano group originating from the imidoyl chloride of the starting material using thermal or reactions with thiophiles. An interesting example of an ANRORC-style mechanism leading to a ring transformation was the reaction of (*Z*)-*N*-(4-chloro-5*H*-1,2,3-dithiazol-5-ylidene)-1*H*-pyrazol-5-amines **4d** with diethylamine that results in disulfide intermediates **18**. Subsequent treatment with concentrated sulfuric acid gave 1,2,4-dithiazines **19** in good yields [43] (Scheme 9).

**Scheme 10:**
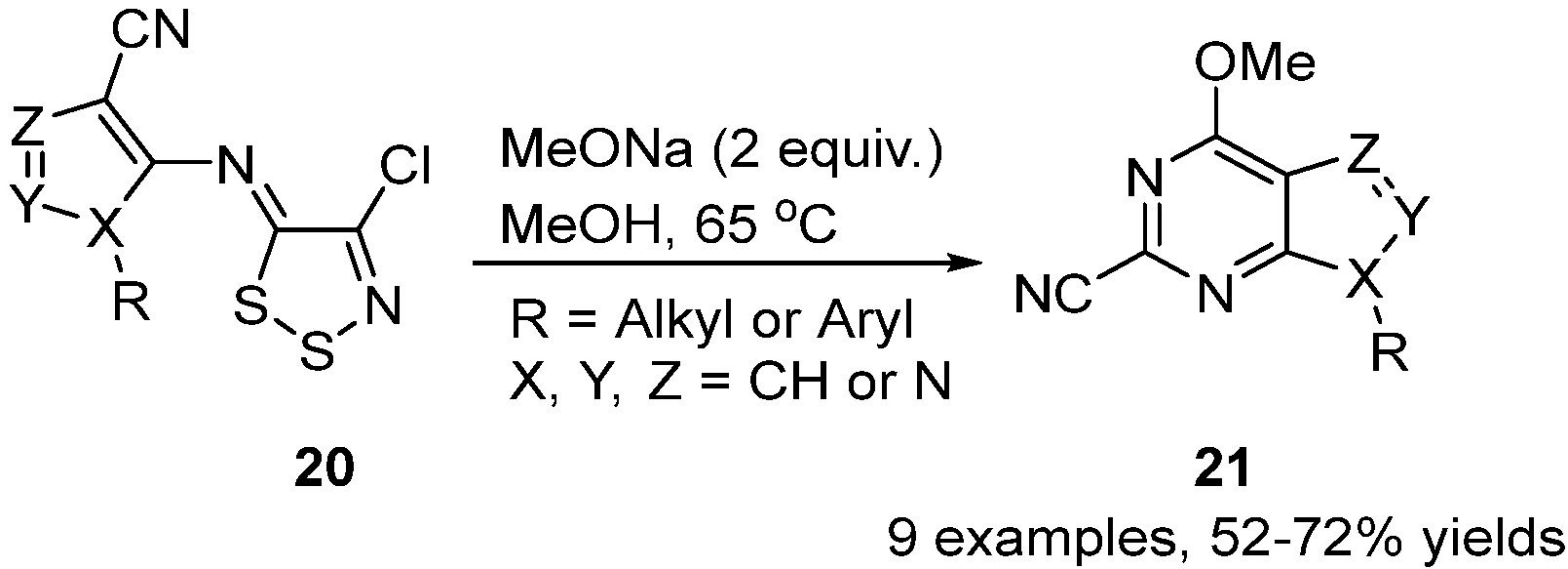
Synthesis of 4-methoxy-pyrazolo[3,4-*d*]pyrimidines **21**.

In another example, the pyrazoleimino dithiazoles **20** were converted to 4-methoxy-pyrazolo[3,4-*d*]pyrimidines **21** in medium to good yields by treatment with sodium methoxide in methanol [16] (Scheme 10). The transformation occurs after addition of the methoxide on the nitrile followed by cyclisation onto the dithiazole C5 position that fragments losing S_2_ and chloride to give the final pyrimidine **21**.

**Scheme 11:**
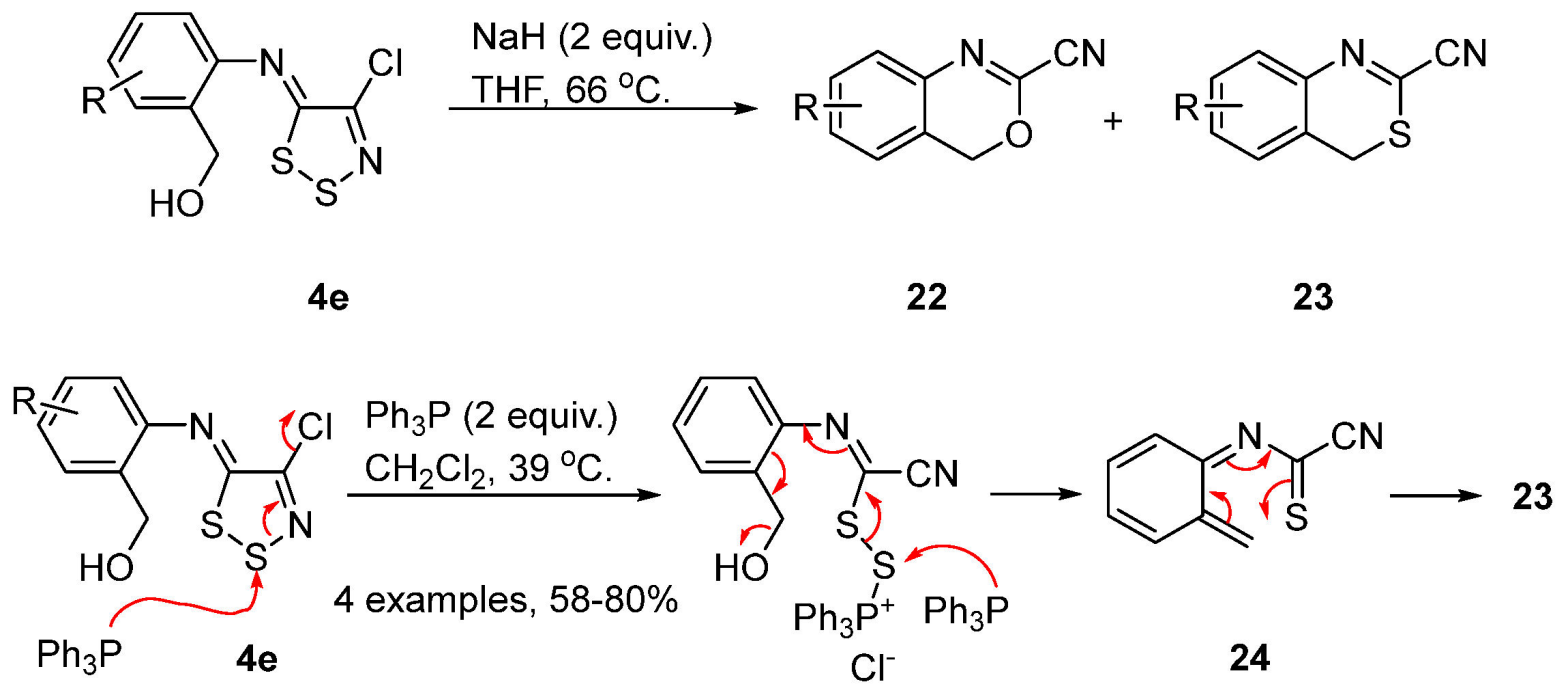
Synthesis of 1,3-benzoxazines **22** and 1,3-benzothiazines **23**.

A similar example of ring transformations is that of 2-aminobenzyl alcohol dithiazole-imines **4e** to 1,3-benzoxazines **22** and 1,3-benzothiazines **23 [44]**. Treatment of imines **4e** with sodium hydride in THF gave mixtures of benzoxazines **22** and benzothiazines **23**, with the former as the main products (Scheme 11). The formation of the former involves deprotonation of the alcohol and cyclisation of the alkoxide onto the dithiazole C5 position. Subsequent fragmentation with loss of S_2_ and chloride gave the final benzoxazine **22**. Alternatively, treatment of imines **4e** with Ph_3_P gave exclusively benzothiazines **23** in good yields (Scheme 11). Thiophilic attack on S1 ring opens the dithiazole ring and a second attack by Ph_3_P gives the intermediate alkene **24** that cyclizes to benzothiazine **23**.

**Scheme 12:**
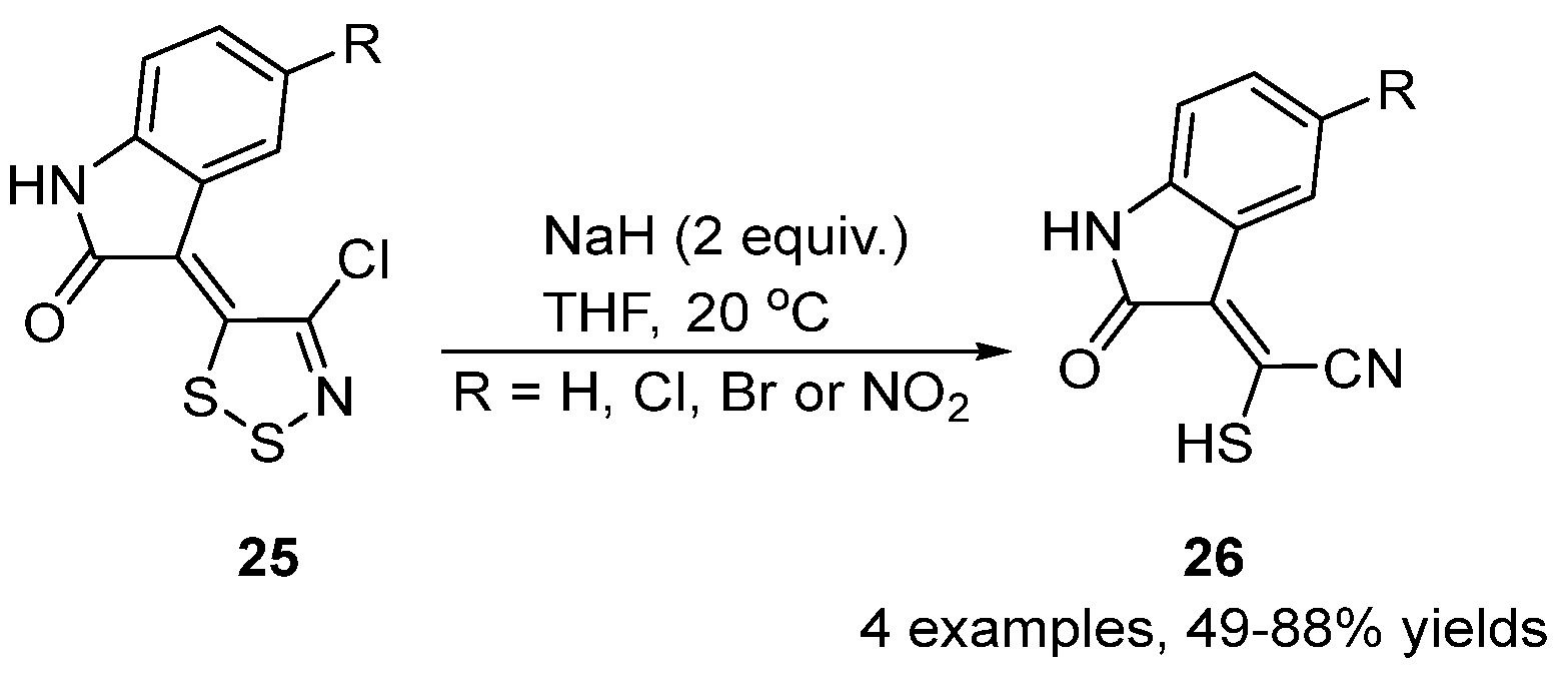
Conversion of dithiazoles **25** to mercaptoacetonitriles **26**.

1,2,3-Dithiazole derivatives can also be converted to mercaptoacetonitriles by the removal of the S1 atom. One example of this are the 3-(1,2,3-dithiazolylidene)indololin-2-ones **25** reacting with sodium hydride (2 equiv.) to yield the mercaptoacetonitrile products **26** in medium to good yields [45] (Scheme 12).

**Scheme 13:**
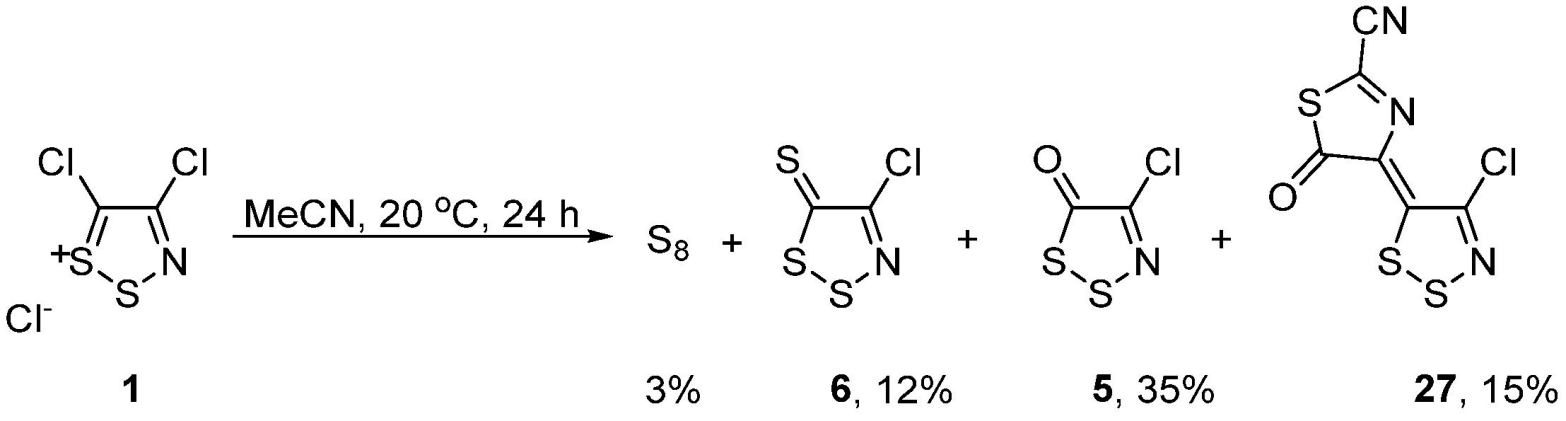
Degradation of Appel salt **1** in wet MeCN.

Perhaps the most unstable 1,2,3-dithiazole is Appel salt itself, which, while relatively stable at ca. 20 °C under a desiccant, in its absence, Appel salt has a tendency to react with moisture. One study by Koutentis et al. revealed that simple stirring in wet MeCN gave elemental sulfur, dithiazole-5-thione **6**, dithiazol-5-one **5,** and thiazol-5-one **27** [46] (Scheme 13), assisting other scientists working with Appel salt, to identify these products. Interestingly, other dithiazolium salts have also been prepared with increased stability and lower sensitivity to moisture. A series of perchlorate salts of 1,2,3-dithiazoles were prepared by the anion exchange with perchloric acid allowing for more detailed characterization and study of the 1,2,3-dithiazole [29].

**Scheme 14:**
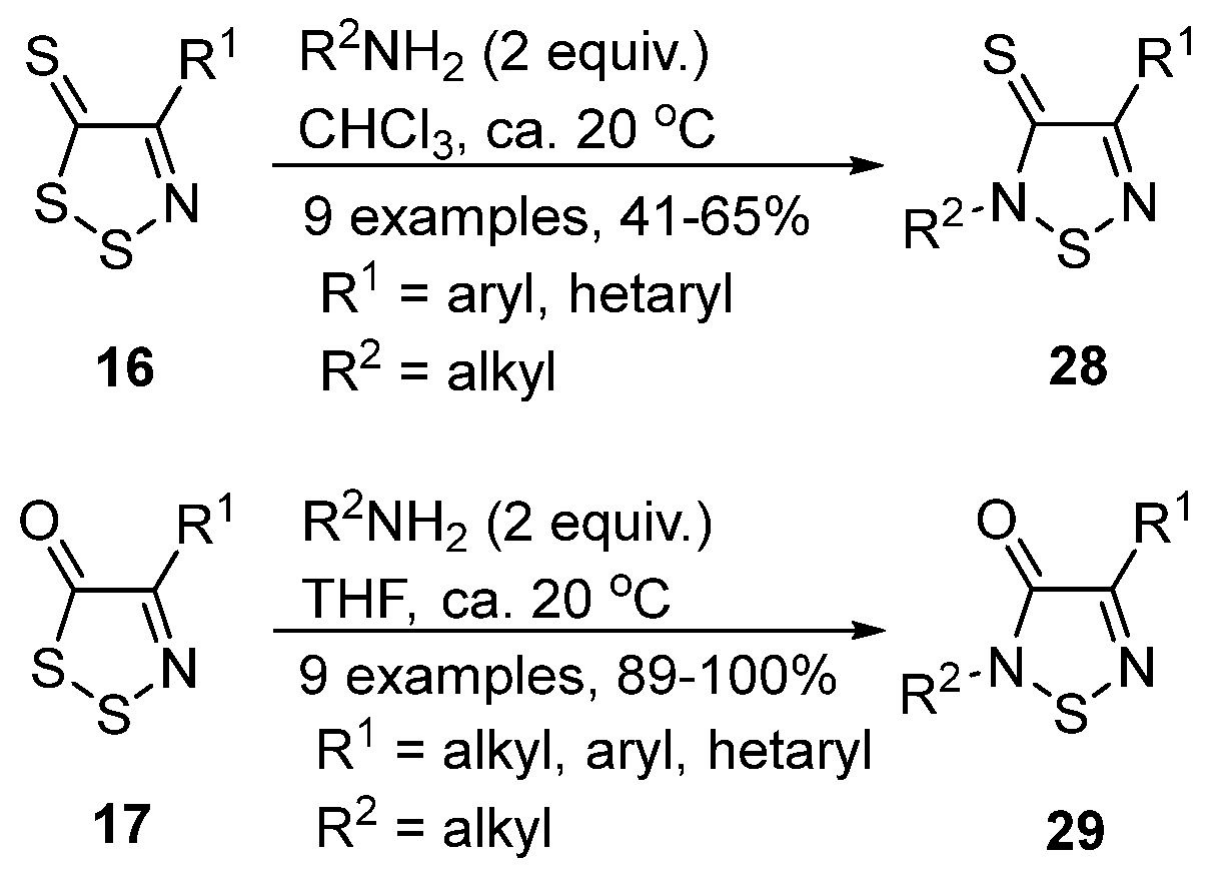
Transformation of dithiazoles **16**–**17** to thiadiazines **28**–**29**.

In another study by Rakitin et al., 4-substituted 5*H*-1,2,3-dithiazoles **16** and **17** were converted to 1,2,5-thiadiazoles **28** and **29** by treatment with primary amines [47] (Scheme 14). Mechanistically, the reaction occurs by addition of the amine to the C5 position followed by ring opening of the C-S bond and subsequent ring closing by loss of hydrogen sulfide.

**Scheme 15:**
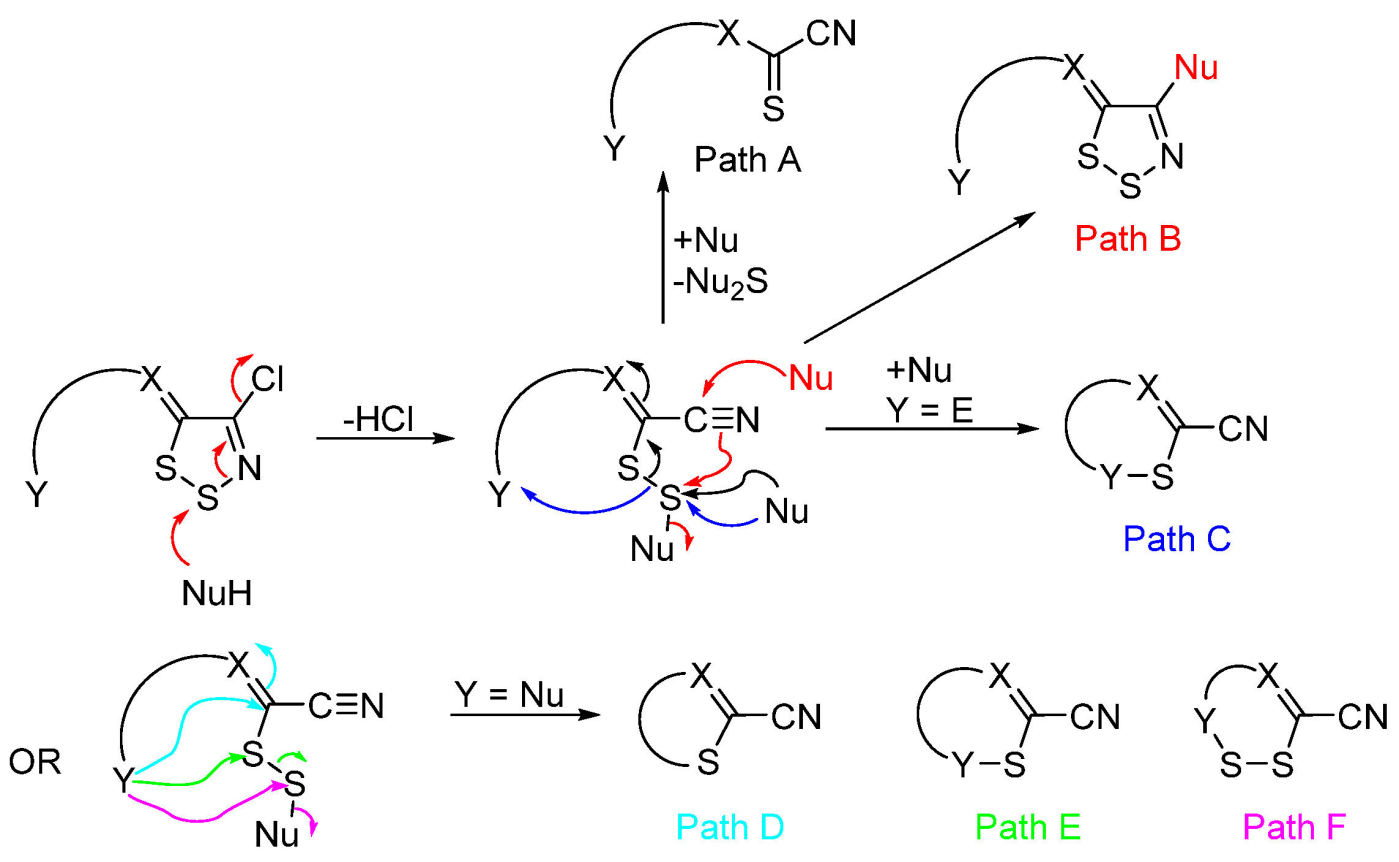
Overview of the mechanisms of the reactions of 1,2,3-dithiazoles.

To summarize, 1,2,3-dithiazoles can be converted to other heterocyclic or ring opened derivatives. The six most common mechanisms involved in the transformations of 1,2,3-dithiazoles to other systems are shown below (Scheme 15). These mechanisms begin *via* a nucleophile assisted ring opening of the dithiazole to disulfide intermediates that then can react either intermolecular or intramolecular with other nucleophiles *via* the six paths presented.

## 3. 1,2,3-Dithiazoles in Medicinal Chemistry

### 3.1. Antimicrobial Activities of 1,2,3-Dithiazoles, including Antifungal, Herbicidal, and Antibacterial 

The first report of biological activity using the 1,2,3-dithiazole scaffold was published in a patent filed by J. E. Moore in 1977 on behalf of Chevron Research Co. [2,3]. The patent disclosed a series of novel 1,2,3-dithiazoles afforded in a 2–3 step sequence from *N*-aryl cyanothioformamide and sulfur dichloride. The main application of these compounds was the controlling of various fungal infections, leaf blights, invasive plant species, and mites.

**Figure 2:**
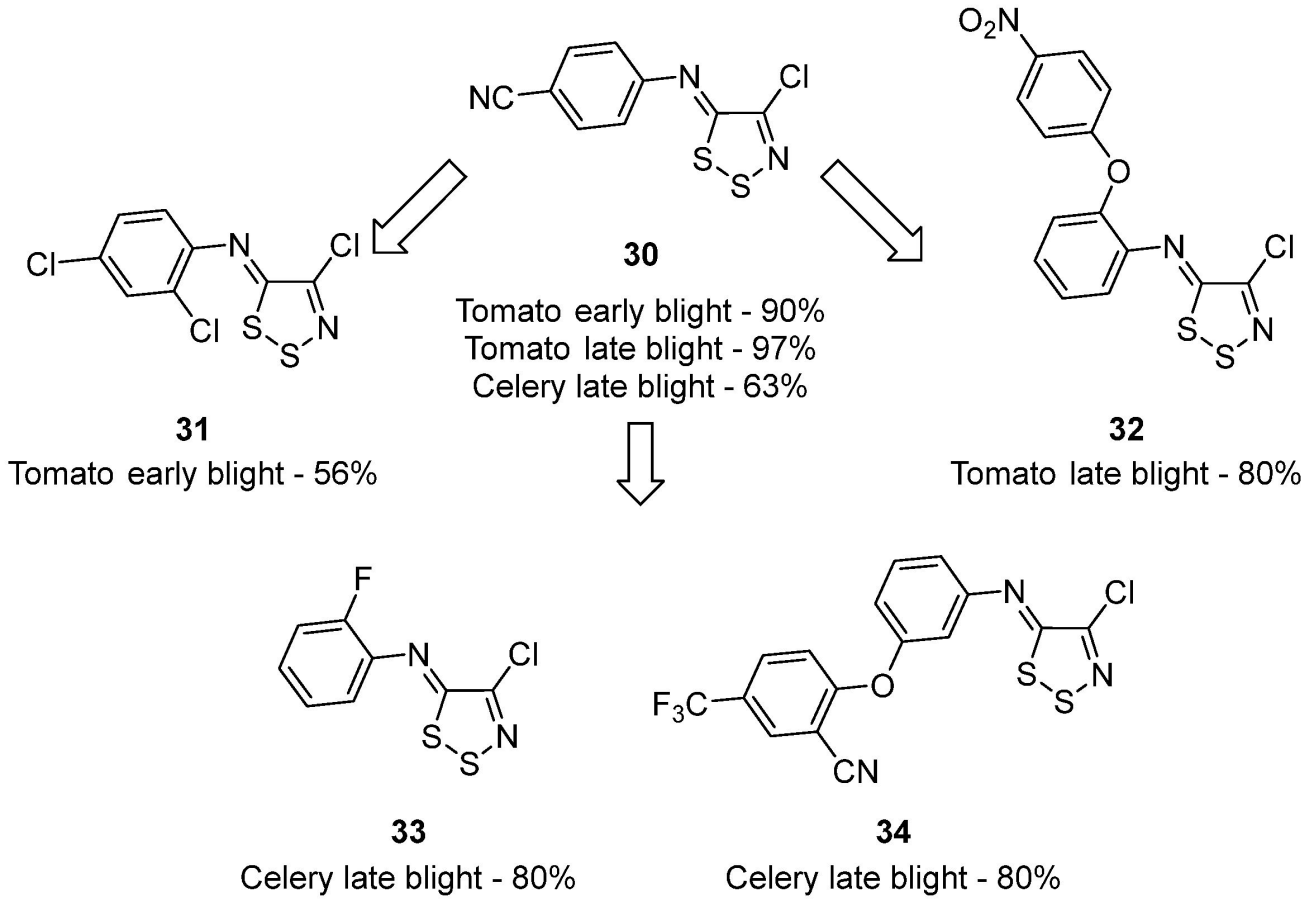
Antifungicidal activities of early 1,2,3-dithiazole derivatives at a concentration of 250 ppm.

First, the tomato early blight organism, *Alternaria solani conidia* was tested against 6- to 7-week-old tomato plate seedlings. The tomato plants were sprayed with 250 ppm solutions of a 1,2,3-dithiazole library. This resulted in the identification of (Z)-4-((4-chloro-5*H*-1,2,3-dithiazol-5-ylidene)amino)benzonitrile (**30**) with a 90% reduction compared with non-treatment. The 2,4-dichloro analogue **31** had weaker activity, with a reduction of just over half of the infection (Figure 1). Next, the tomato late blight organism, *Phytophthora infestans conidia* was tested against seedlings of 5 to 6 weeks old using the same procedure. The 4-cyano analogue **30** was found to afford 97% protection, while the 2-(4-nitrophenoxy) analogue **32** showed an 80% reduction (Figure 2). Then, the celery late blight organism *septoria api* was tested using 11-week-old plants. The 4-cyano analogue **30** afforded less protection at just over 60%, while several other analogues showed improvements, including 2-fluoro **33** and 3-(4-trifluoro, 2-cyanopenoxy) **34** analogues, both reported with 80% protection (Figure 2).

**Figure 3:**
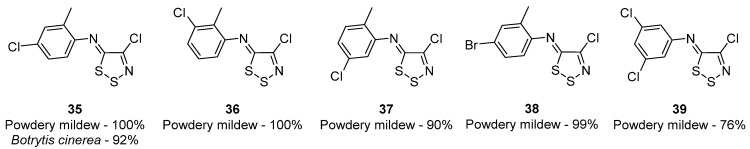
Antifungicidal activities of 1,2,3-dithiazoles against powdery mildew (250 ppm) and *Botriytis cinerea* (40 ppm).

A series of halogenated analogues **35**–**39** were then identified as active against the powdery mildew pathogen *Erysiphe polygoni* using bean seedlings with well-developed primary leaves. The (Z)-4-chloro-*N*-(4-chloro-2-methylphenyl)-5*H*-1,2,3-dithiazol-5-imine analogue (**35**) along with the corresponding 3-chloro **36** showed 100% protection at 250 ppm. The corresponding 5-chloro **37** and 4-bromo **38** both showed a small reduction in efficacy, 10% and 1%, respectively, while the 3,5-dichloro **39** was only net 76% effective (Figure 3).

**Figure 4:**
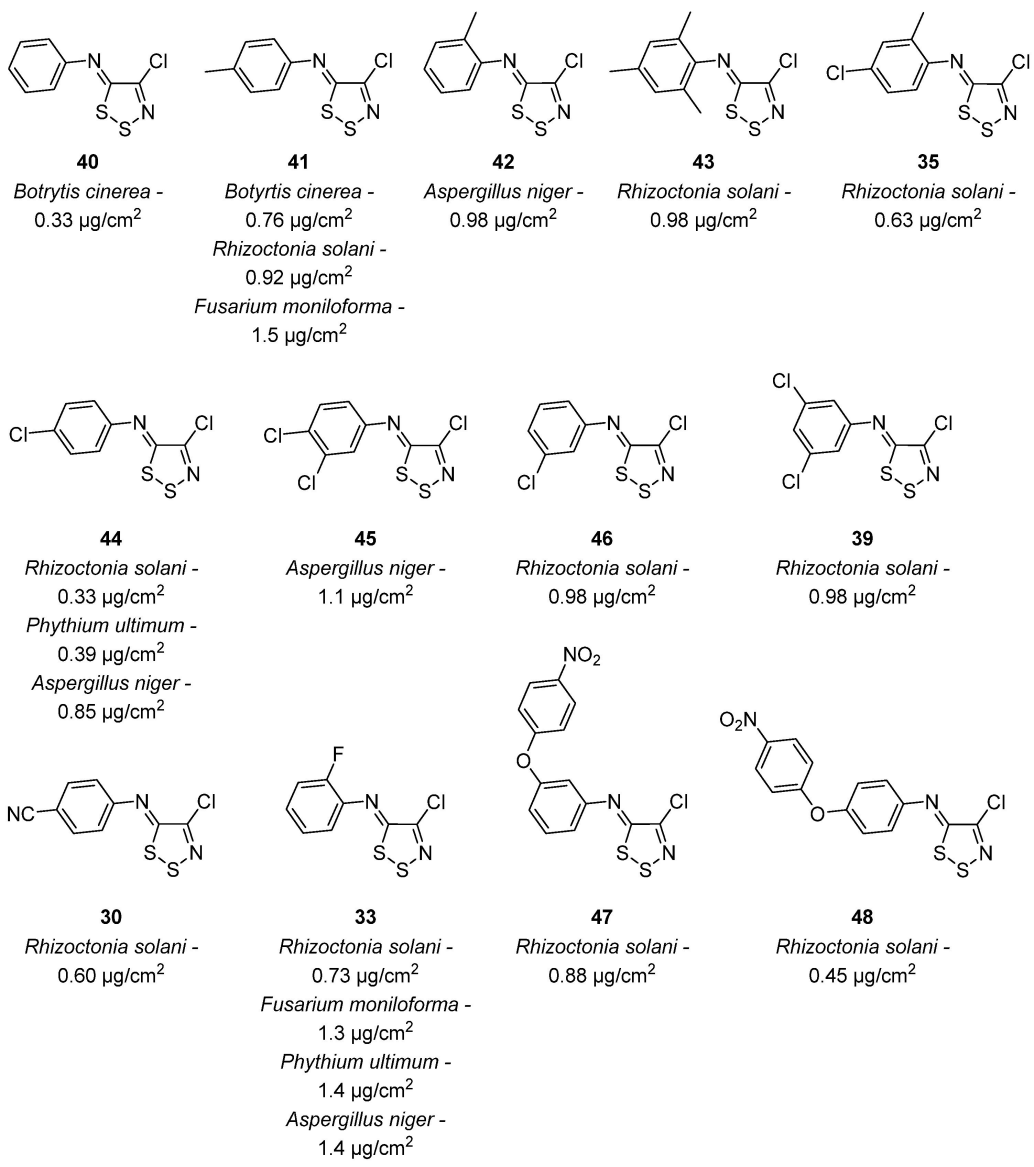
Antifungicidal activities of 1,2,3-dithiazoles, values are amounts required for mycelia inhibition, micrograms/cm^2^ for 99% control of fungal growth.

**Figure 5:**
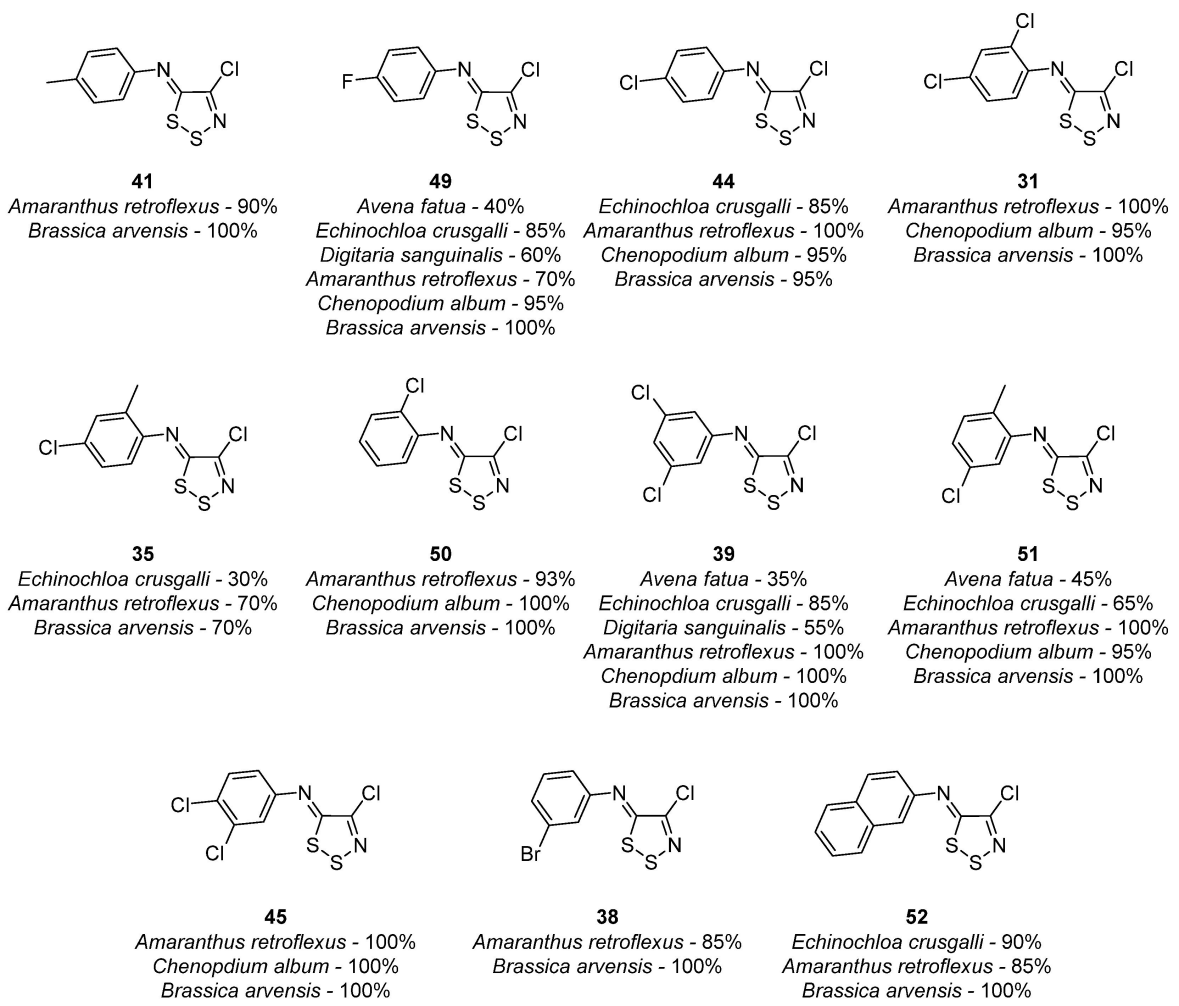
Herbicidal activities of early 1,2,3-dithiazole derivatives tested at 33 ppm.

Initial screening was also carried out against necrotrophic fungus *Botrytis cinerea* on the well-developed primary leaves of a 4–6-week-old horsebean plant at a lower concentration (40 ppm). Only 1,2,3-dithiazole **35** was demonstrated to be effective with 92% inhibition (Figure 3). However, after this initial result, screening was carried out on a broader panel of fungal (Figure 4) and herbicidal strains (Figure 5). The fungal panel included *Botrytis cinerea*, *Rhizoctonia solani*, *Fusarium moniloforma*, *Phythium ultimum,* and *Aspergillus niger*. The compounds **30**, **33**, and **39**–**48** were tested at 500 ppm and fungicidal activities were measured by the zone of inhibited mycelia growth (Figure 4). Interestingly, the unsubstituted phenyl analogue (*Z*)-4-chloro-*N*-phenyl-5*H*-1,2,3-dithiazol-5-imine (**40**) was active on *Botrytis cinerea* at 0.33 μg/cm^2^. The addition of a 4-position methyl in analogue **41** reduced the activity against *Botrytis cinerea* by over 2-fold, but increased the activity against *Rhizoctonia solani* and *Fusarium moniloforma*. The 2-position methyl analogue **42** showed a profile switch showing activity only against *Aspergillus niger* (0.98 μg/cm^2^). The 2,4,6-trimethyl analogue **43** also only retained activity against on strain *Rhizoctonia solani* (0.98 μg/cm^2^). The original 4-chloro, 2-methyl analogue **35** showed activity against *Rhizoctonia solani* (0.63 μg/cm^2^), but the dose dependent *Botrytis cinerea* data was not reported. The removal of the methyl group to afford the 4-chloro analogue **44** increased the activity against *Rhizoctonia solani* by 2-fold and showed commensurate activity against *Phythium ultimum* and 3-fold weaker activity against *Aspergillus niger*. The addition of a second chloro in the 3-position in analogue **45** was unfavored with only activity against *Aspergillus niger* retained. When the 4-chloro is removed to afford **46**, the activity is switched again with potency only demonstrated for *Rhizoctonia solani* at the same level as **43**. Addition of a second choro at the 5-position to afford **47** has same activity profile as **46**. The 4-cyano analogue **30** showed good activity against *Rhizoctonia solani* (0.60 μg/cm^2^). The 2-fluoro analogue **33**, while having a slightly weaker potency, did show activity against 4 out of 5 of the fungal panel, only excluding *Botrytis cinerea.* The final two analogues identified in this series, 3-(4-nitrophenoxy) **47** and 4-(4-nitrophenoxy) **48**, both showed activity against only *Rhizoctonia solani* with analogue **48** having a 2-fold improvement over **47** at (0.45 μg/cm^2^).

The 1,2,3-dithiazoles were then screened at 33 ppm on a herbal panel that included wild oats (*Awena fatua*), watergrass (*Echinochloa crusgall*), crabgrass (*Digitaria sanguinalis*), mustard (*Brassica arversis*), pigweed (*Amaranthus retroflexus*), and lambsquarter (*Cheropodium album*) (Figure 5). The first analogue (*Z*)-4-chloro-*N*-(*p*-tolyl)-5*H*-1,2,3-dithiazol-5-imine (**41**), showed good efficacy against *Amaranthus retroflexus* (90%) and total control of *Brassica arvensis*. Switching to the 4-fluoro analogue **49** increased coverage across all strains tested, including *Avena fatua* (40%), which was only weakly inhibited across the series and total control of *Amaranthus retroflexus.* The 4-chloro analogue **44** was 3-fold less effective against *Digitaria sanguinalis* and *Avena fatua*. The addition of a 2-position chloro **50** decreased strain coverage, but did mean total control of *Brassica arvensis* in addition to *Amaranthus retroflexus*, with additional high efficacy against *Chenopodium Album* (95%). The original 2-methyl 4-chloro analogue **35**, while still showing efficacy across several strains, did not offer total or near total control for any of the strains tested. The 2-chloro analogue **50** showed total control for *Chenopodium album* and *Brassica arvensis* and near total for *Amaranthus retroflexus* (93%). However, 2-chloro **50** had a limited effect on *Digitaria sanguinalis* and *Echinochloa crusgalli*, with no impact on *Avena fatua*. The 3,5-dichloro analogue **39** demonstrated good efficacy against most strains, including total control of *Amaranthus retroflexus*, *Chenopdium album, Brassica arvensis,* and some activity against *Avena fatua* (35%). The 2-methyl, 5-chloro analogue **51** offered the highest efficacy across the series on *Avena fatua* (45%), total control *of Amaranthus retroflexus* and *Brassica arvensis*, with near total control of *Chenopodium album* (95%). The 3,4-dichloro analogue **45** had a potent but narrower band of activity with total control of *Amaranthus retroflexus*, *Chenopdium album* and *Brassica arvensis*, but weaker activity on the other three strains (30–55%). The 3-bromo analogue **38** has a similar profile to the 4-methyl **41**, while the 2-naphthyl analogue **52** was the most potent in the screening for *Echinochloa crusgalli* (90%) and offered good control over *Amaranthus retroflexus* (85%) and total control over *Brassica arvensis*.

**Figure 6:**
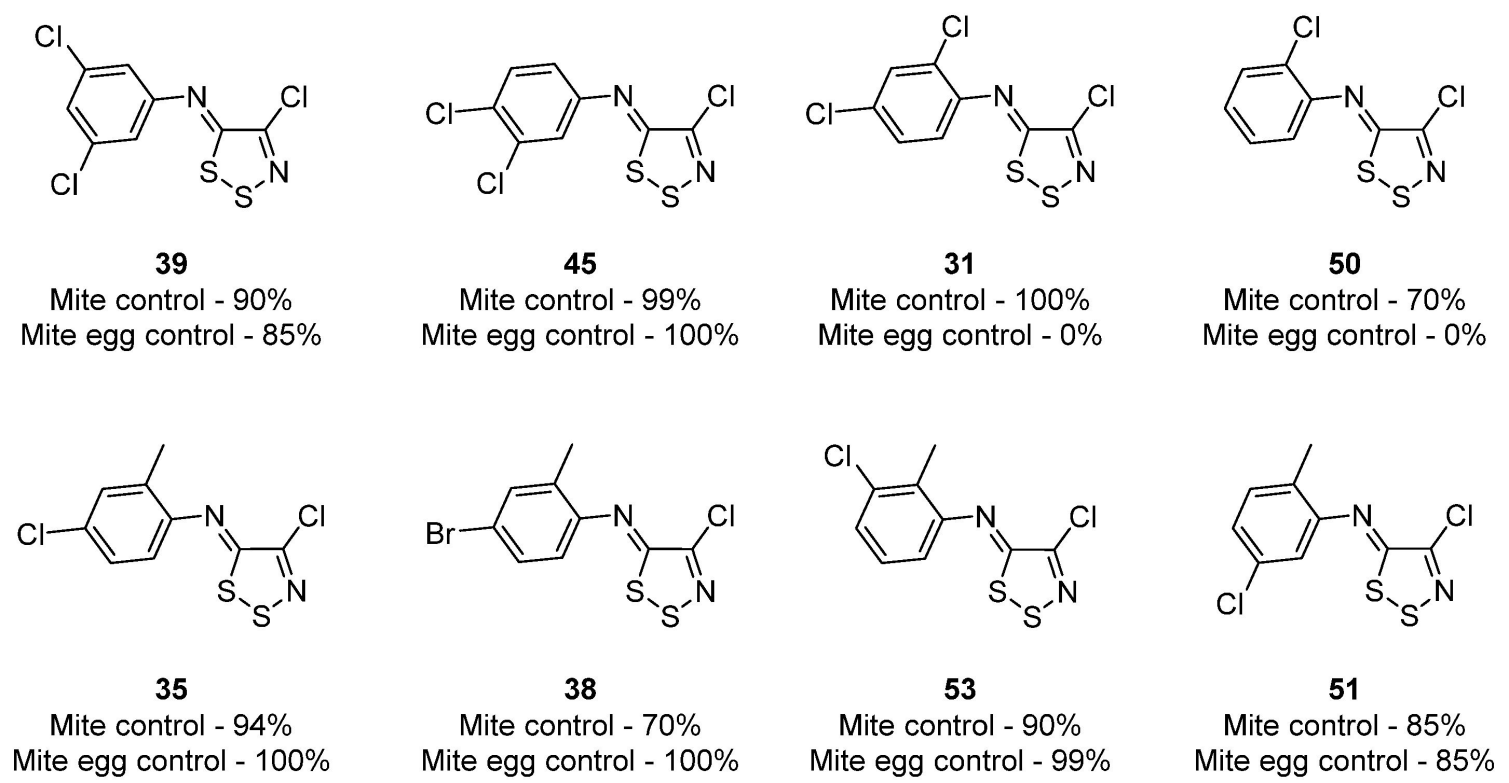
Mite (*Tetranychus urticae*) control activities of early 1,2,3-dithiazole derivatives at 40 ppm.

In order to test for other pests, pinto bean leaves were treated with two spotted mites (*Tetramuchus urticae*). The mites were then allowed to lay eggs on the leaves, and after 48 h, the leaves were treated with 40 ppm of the test compound (Figure 6). A series of halogenated phenyl-5*H*-1,2,3-dithiazol-5-imines were identified with activity against both *Tetramuchus urticae* and their eggs. The 3,5-dichloro analogue **39** showed a high degree of control with 90% of mites and 85% of eggs suppressed. This increased to almost total control with the 3,4-dichloro **45**. Interestingly, the 2,4-dichloro analogue **31** demonstrated total mite control but had no effect on the eggs. The mono-substituted 2-chloro analogue had a similar profile with no effect on the mite eggs, but only 70% effective control of the mite. The 4-chloro, 2-methyl analogue **35** showed complete egg control and almost complete mite control (94%). The switch to the bromo **38** showed a similar profile, but with 70% mite control. The 2-methyl substituted match pair analogues 3-chloro **56** and 5-chloro **52** both demonstrated a high level of mite and egg control with the 3-position preferred.

Subsequent to the work reported by Chevron Research Co., in 1980, a brief patent was filed by Appel, R. et al. on behalf of Bayer AG on the use of 1,2,3-dithiazoles as antifungals specifically against *Trichophyton Mentagrophytes* [48]. This was followed up by another brief patent in 1984 by Mayer R. et al. on behalf of Dresden University of Technology (Technische Universität Dresden) on the use of *N*-arylcyanothioformamides derived from 1,2,3-dithiazoles as herbicides and crop protection agents [49].

**Figure 7:**
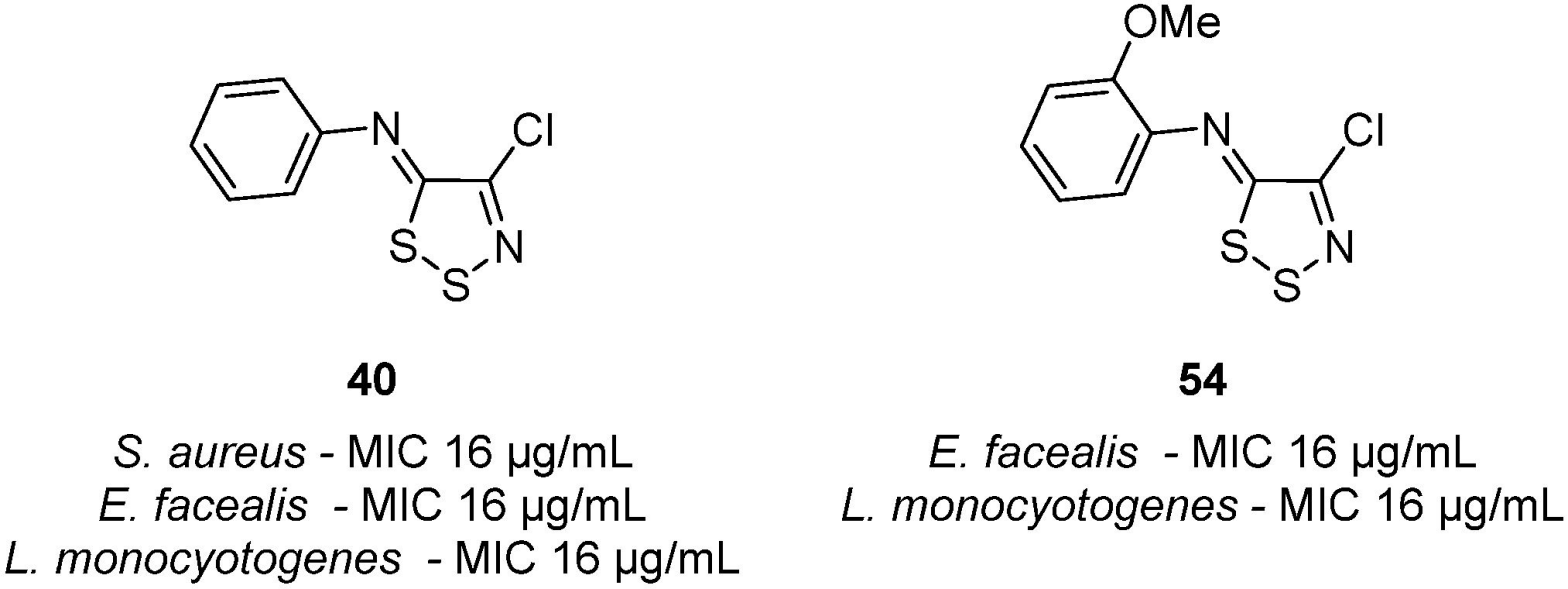
Report on a small panel of 1,2,3-dithiazoles highlighted some nascent antibacterial activity on the dithiazole scaffold.

The 1,2,3-dithazoles chemical space and synthesis progressed as outlined in Section 2.2 during the late 1980s and early 1990s. However, it was not until 1996 when Pons et al. disclosed a focused series of *N*-arylimino-1,2,3-dithiazoles and related *N*-arylcyanothioformamides before further biology was elucidated [15]. The unsubstituted aromatic compound **40** and the 2-methoxy analogue **54** were shown to have potent activity on several bacteria strains (Figure 7). Compound **40** had an MIC of 16 μg/mL on *S. aureus*, *E. faecalis*, and *L. monocyotogenes*, while 2-methoxy **54** had the same level of potency, but only on *E. faecalis* and *L. monocyotogenes*. Interestingly, all the *N*-arylcyanothioformamides analogues tested were ineffective, highlighting the need for the 1,2,3-dithiazole ring.

**Figure 8:**
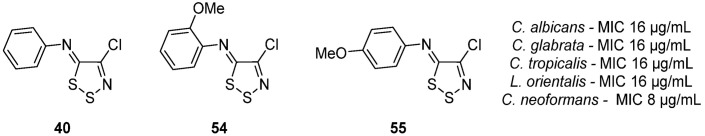
Report on a small panel of 1,2,3-dithiazoles highlighted some nascent antifungal activity on the dithiazole scaffold.

This work was extended in a subsequent report by Pons et al. [50], where a focused library of 1,2,3-dithiazoles and related analogues were screened on a series of fungal targets. The 1,2,3-dithiazoles were the only compounds that showed antifungal activity, with most potent analogues identified as unsubstituted aromatic **40**, the 2-methoxy **54**, and 4-methoxy analogue **55** (Figure 8). These three most potent analogues all had an MIC of 16 μg/mL on *C. albicans*, *C. glabrata*, *C. tropicalis*, *L. orientalis,* and an MIC of 8 μg/mL on *C. neoformans*.

**Figure 9:**
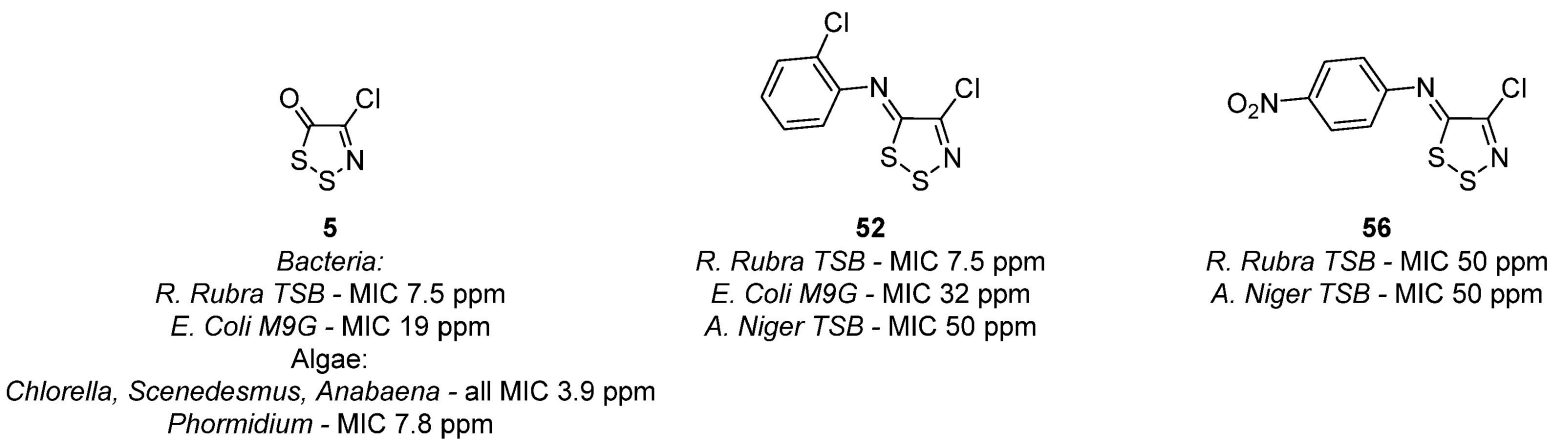
Rohm & Haas Co. filed a patent for industrial applications around three 1,2,3-dithiazoles for antibacterial and antialgae properties.

This was followed by a patent filed in 1997 by Joseph, R. W. et al. on behalf of Rohm & Haas Co. [51], a company specializing in the manufacture of coatings. The disclosed innovation involved the use of 1,2,3-dithiazoles to rapidly inhibit microbial and algae growth for industrial applications. These included paints, coatings, treatments, and textiles, among others. The effective amount applied was between 0.1 to 300 ppm, with three main exemplar 1,2,3-dithiazoles highlighted (Figure 9). This included 4-chloro-5*H*-1,2,3-dithiazol-5-one (**5**) with potent antibacterial properties against *R. Rubra TSB* (MIC = 7.5 ppm) and *E. Coli M9G* (MIC = 19 ppm), with potent algae inhibition of *Chlorella*, *Scenedesmus,* and *Anabaena* (all MIC = 3.9 ppm) and *Phormidium* (MIC = 7.8 ppm). In addition to **5,** the 2-chloro analogue **52** was reported to have potent activity against *R. Rubra TSB* (MIC = 7.5 ppm) and good activity against *E. Coli M9G* (MIC = 32 ppm) and *A. Niger TSB* (MIC = 50 ppm). The 4-nitro analogue **56** also performed well with both *E. Coli M9G* and *A. Niger TSB* having an MIC or 50 ppm. The activity reported between **5** and **52** on *E. Coli M9G* is the first evidence of activity against a Gram-negative bacterium. The company also provided data with time of addition experiments showing that 5 and 10 ppm of **5** are effective at 1 h, whereas 10 ppm of methylene *bis*thiocyanate (MBT), a known commercial antimicrobial compound, is not effective until 24 h.

**Figure 10:**
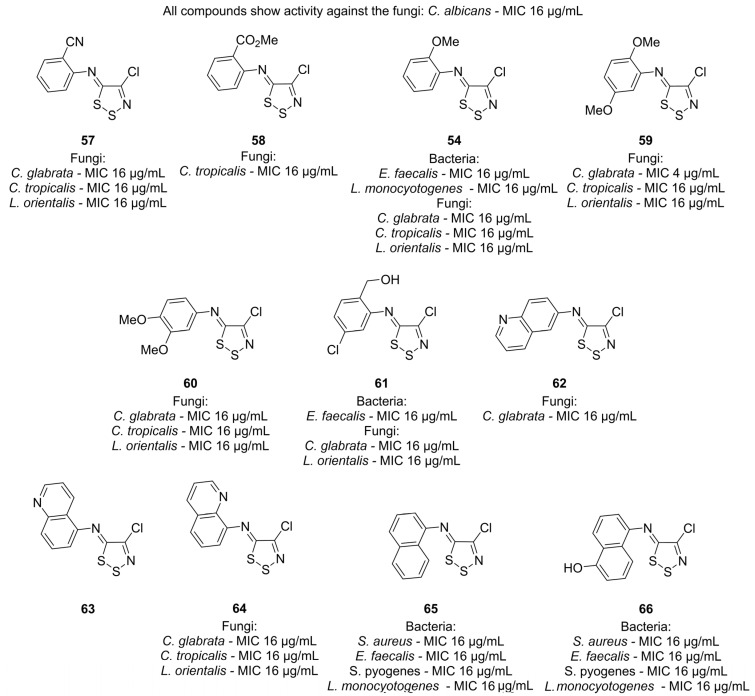
Results of a focused investigation of antibacterial and antifungal activities of selected 1,2,3-dithiazoles [52,53].

Subsequently in 1998, more detailed screening and structure activity relationships (SAR) were published from Pons et al. related to the antimicrobial properties of the 1,2,3-dithiazole scaffold [52,53]. These two studies tested activity against bacteria: *S. aureus*, *E. faecalis*, *S. pyogenes*, and *L. monocytogenes*, and fungi: *C. albicans*, *C. glabrata*, *C. tropicalis,* and *I. orientalis*. This screening supported earlier work on the 1,2,3-dithiazole scaffold, and broadened the scope of this inhibition to several new fungal and bacteria strains (Figure 10). The compounds showed antibacterial activity against Gram-positive bacteria, but as previously described [15], there was no activity against Gram-negative bacteria.

The unsubstituted analogue **40** was a direct repeat of all activities previously demonstrated with antibacterial *S. aureus*; *E. faecalis*; and *L. monocyotogenes* (all MIC = 16 μg/mL); and antifungal *C. albicans*, *C. glabrata*; *C. tropicalis*; and *L. orientalis* (all MIC = 16 μg/mL). All of the highlighted compounds (**40**, **54**, **57–66**) had *C. albicans* activity at MIC = 16 μg/mL. The 2-cyano analogue **57** had activity (MIC = 16 μg/mL) across all fungal strains tested but had limited antibacterial effects. Switching to the 2-methylester **58** narrowed the antifungal activity. However, the 2-methoxy **54** had good broad spectrum antimicrobial activity hitting 7 out of the 8 strains tested. The introduction of a second methoxy group in the 5-position to afford (*Z*)-4-chloro-*N*-(2,5-dimethoxyphenyl)-5*H*-1,2,3-dithiazol-5-imine (**59**) increased the potency (MIC = 4 μg/mL) on *C. glabrata*, while maintaining antifungal coverage. Moving the 2-position methoxy to the 4-position in analogue **60** maintained the antifungal coverage but lost the 4-fold boost seen against *C. glabrata* with **59**. The (*Z*)-(4-chloro-2-((4-chloro-5*H*-1,2,3-dithiazol-5-ylidene)amino)phenyl)methanol (**61**) analogue showed potency against *E. faecalis*, *C. glabrata*, *C. albicans,* and *L. orientalis* (all MIC = 16 μg/mL); in addition to demonstrating a tolerability for more diverse substitution patterns.

A switch to fused heterocycles including quinolines and naphthalene was maintained rather than increased overall potency and coverage. The quinolin-6-yl substituted analogue **62** showed potency against *C. glabrata* and *C. albicans* (both MIC = 16 μg/mL), while the quinolin-5-yl **63** was only active against the *C. albicans*. The naphthalen-1-yl **65** and hydroxy substituted naphthalen-1-yl **66** had the same profile with coverage against all four bacteria tested (*S. aureus*, *E. faecalis*, *S. pyogenes*, and *L. monocyotogenes* all MIC = 16 μg/mL) and *C. albicans*. The hydroxy substitution of **65** to afford **66** did not provide any potency advantage, but did demonstrate there was an ability to alter physicochemical properties without affecting potency.

**Figure 11:**
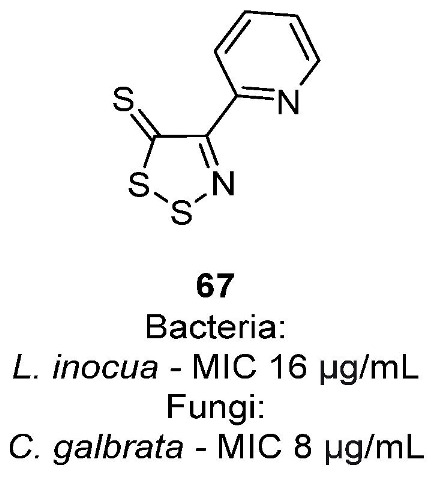
The 4-(pyridin-2-yl)-5*H*-1,2,3-dithiazole-5-thione (**67**) was the only compound with potent antimicrobial activity from the C4 substituted analogue library.

Access to a series of new substituted 5-phenylimino, 5-thieno, or 5-oxo-1,2,3-dithiazoles was reported in 2009 by Rakitin et al. [13] (synthesis discussed in Section 2.4). A series of sixteen compounds were screened against four fungi strains: *C. albicans*, *C. glabrata*, *C. tropicalis*, and *I. orientalis*; four Gram-negative bacteria strains: *E. coli*, *P. aeruginosa*, *K. pneumoniae*, and *S. Typhimurium;* and four Gram-positive bacteria strains: *E. faecalis*, *S. aureus*, *B. cereus*, and *L. inocua*. The result of this screening were some compounds with limited activity and 4-(pyridin-2-yl)-5*H*-1,2,3-dithiazole-5-thione (**67**), which was active against bacteria *L. inocua* (MIC = 16 μg/mL) and fungi *C. galbrata* (MIC = 8 μg/mL) (Figure 11). These results opened up additional chemical space to potentially further investigate the 1,2,3-dithiazole SAR.

**Figure 12:**
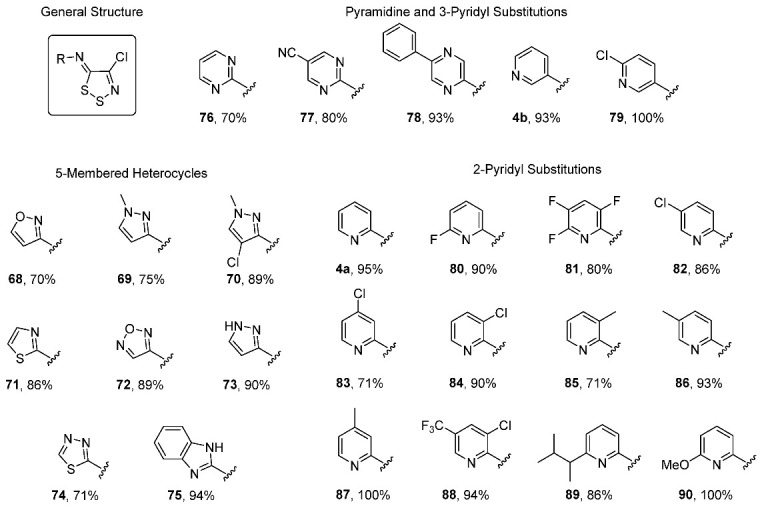
*Phytophtora* (tomato) preventive; ≥70% efficacy at concentration of 1500 ppm.

**Figure 13:**
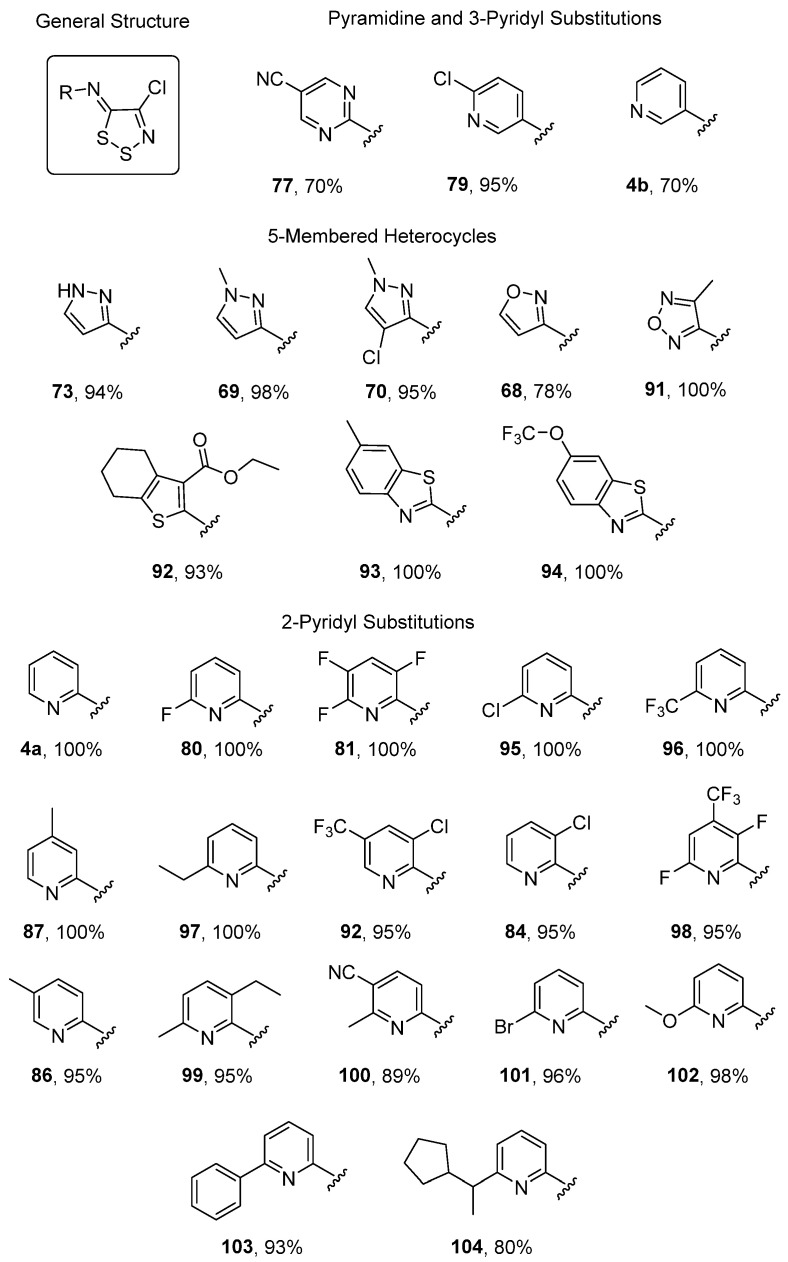
*Sphaerotheca* (cucumber) preventive; ≥70% efficacy at concentration of 1500 ppm.

**Figure 14:**
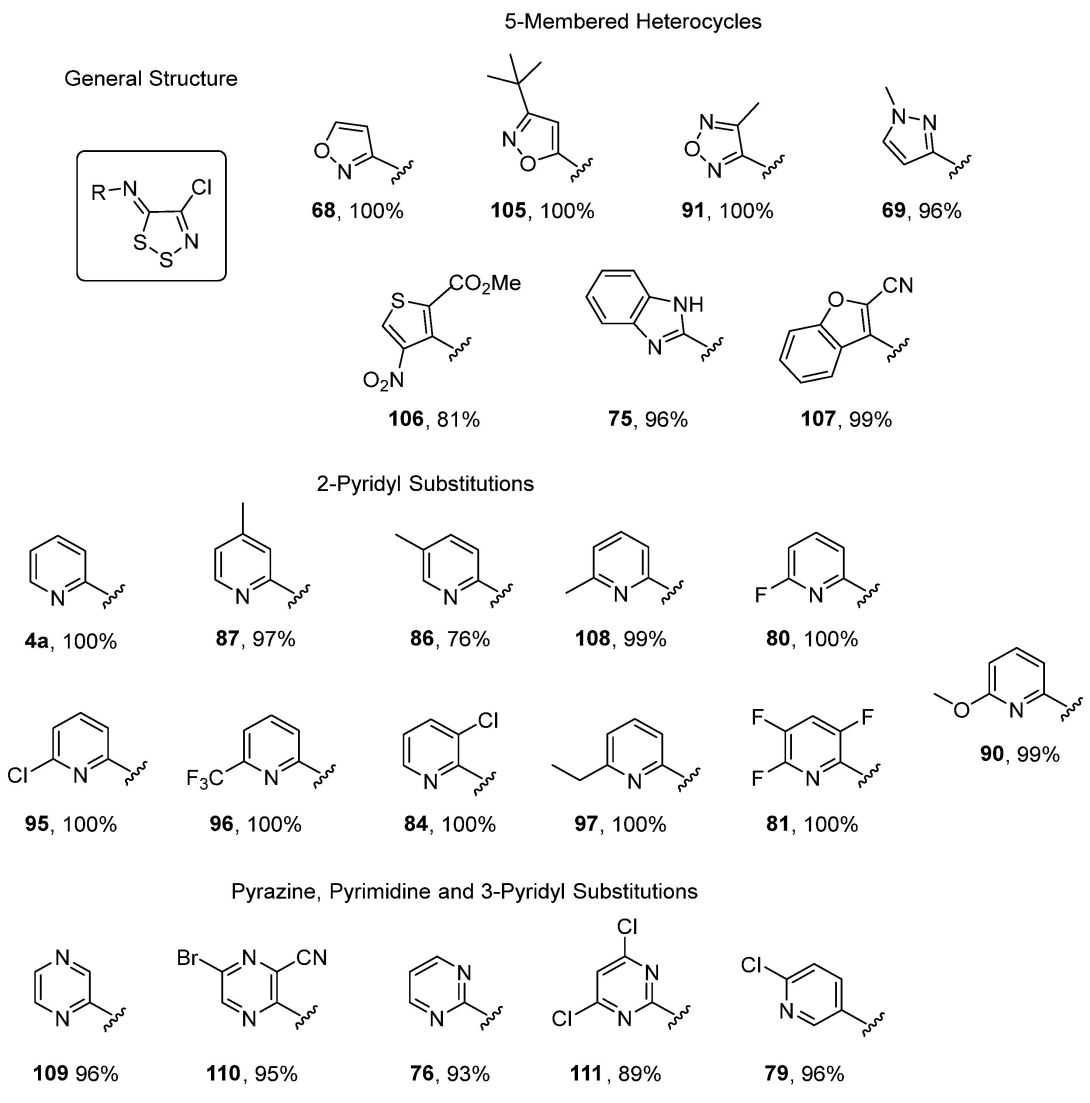
*Venturiatest* (apples) preventive; ≥70% efficacy at concentration of 250 ppm.

**Figure 15:**
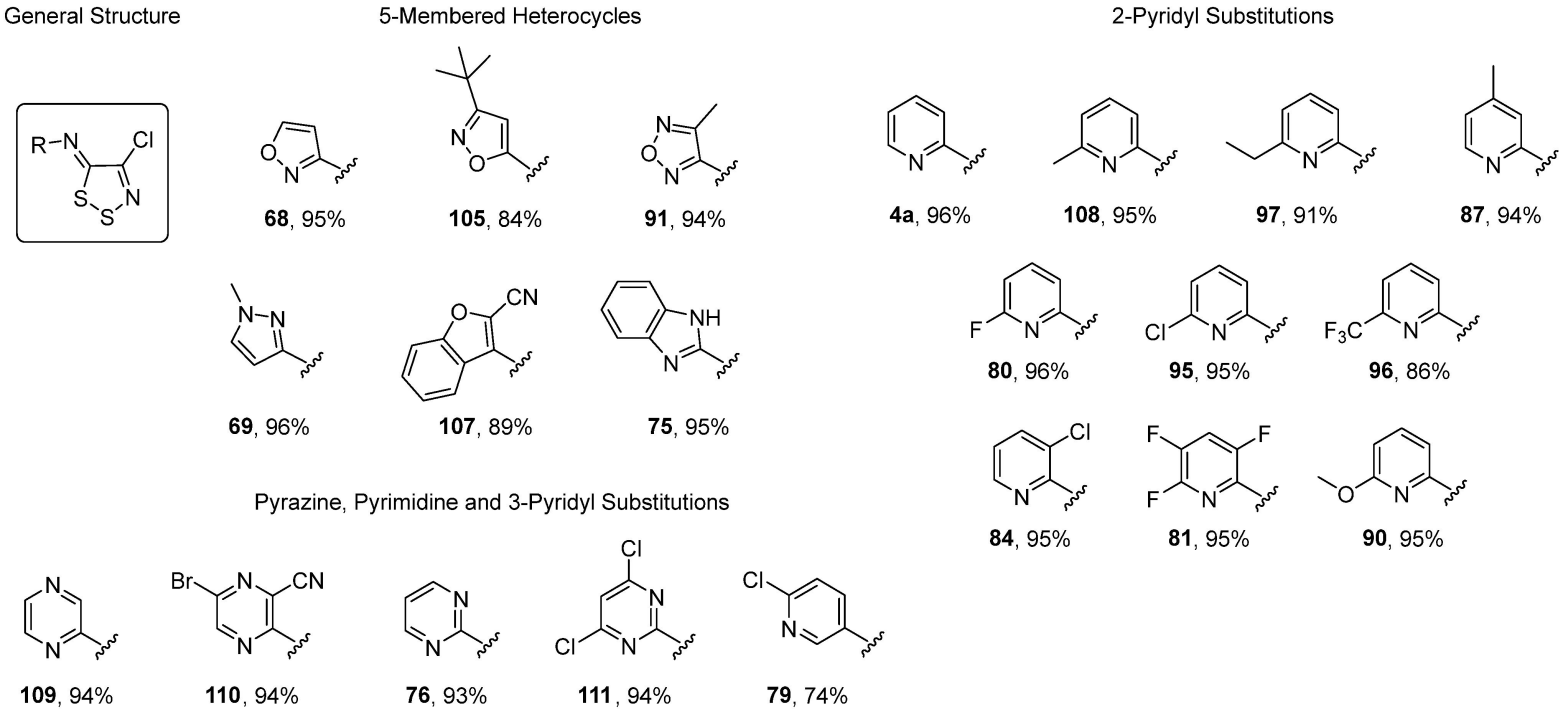
*Alternia* (tomatoes) preventive ≥70% efficacy at concentration of 250 ppm.

**Figure 16:**
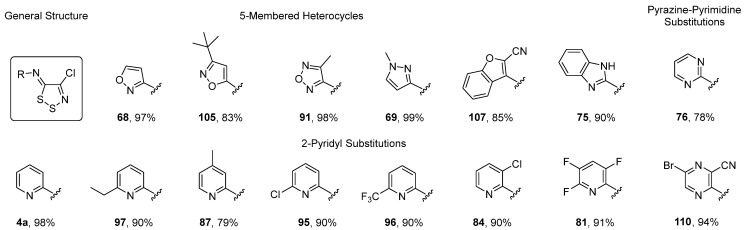
*Botyrtis* (beans) preventive ≥70% efficacy at concentration of 500 ppm.

**Figure 17:**
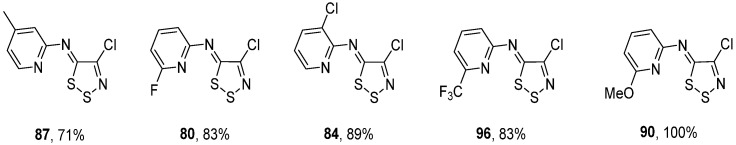
*Leptosphaeria nodorum* (wheat) preventive ≥70% efficacy at concentration of 1000 ppm.

**Figure 18:**
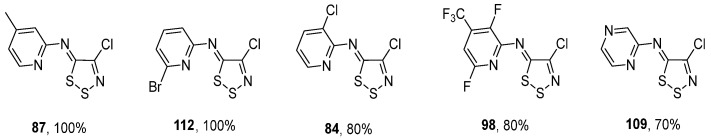
*Septoria tritici* (wheat) preventive ≥70% efficacy at concentration of 1000 ppm.

**Figure 19:**
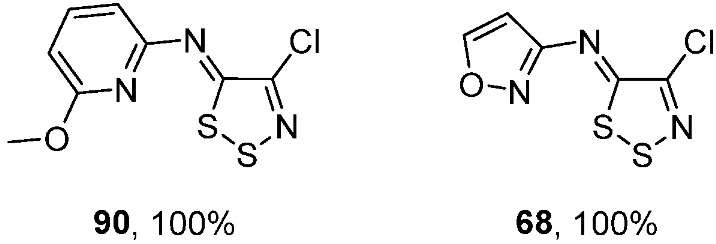
*Pyricularia* (rice) preventive ≥80% efficacy at concentration of 250 ppm.

In 2012, a patent was filed by Benting et al. on behalf of Bayer Cropscience AG focusing on phytopathogenic antifungal crop protection aspects of heteroaromatic substituted 1,2,3-dithiazole analogues [54]. Heteroaromatic substitution patterns had until the point been largely neglected, due in part to electron deficient amines affording lower yields (as in the case of [37]). A library of heteroaromatic 1,2,3-dithiazole derivative were screened against a series of different fungi strains. These included tomato (*Phytophtora*), cucumber (*Sphaerotheca*), apples (*Venturiatest*), tomato (*Alternia*), beans (*Botyrtis*), wheat (*Leptosphaeria nodorum*), wheat (*Septoria tritici*), and rice (*Pyricularia*). Only compounds that showed inhibition above 70% at the respective concentration tested were reported. The results were broadly clustered into three groups, small 5-membered heterocyles, pyrimidine, and 3-pyridyl substituted heterocycles and 2-pyridyl substituted heterocycles. There was broad SAR tolerability for *Phytophtora* (Figure 12), *Sphaerotheca* (Figure 13), *Venturiatest* (Figure 14), *Alternia* (Figure 15), and *Botyrtis* (Figure 16). While with *Leptosphaeria nodorum* (Figure 17), *Septoria tritici* (Figure 18), and *Pyricularia* (Figure 19) the SAR narrowed considerably.

The chemical space around *Phytophtora* inhibition included a broad array of 1,2,3-dithiazol-5-imines **4a** and **68–90** (Figure 12). While most analogues reported were potent; only the 4-methyl 2-pyridyl **87** and 3-methoxy 2-pyridyl **90** achieved total control of the fungi. In the case of *Sphaerotheca*, many compounds demonstrated very high degrees of antifungal control, including **4a**, **4b**, **68–70**, **73**, **77**, **79–81**, **86–87,** and **91–104** (Figure 13), while ten compounds showed complete control including the unsubstituted analogues 2-pyridyl **4a** and 4-methyl 2-pyridyl **91**.

The *Venturiatest* fungi appears to be easier to target as the reported dose is 6-fold less (250 ppm vs. 1500 ppm), and most reported compounds **4a**, **68**–**69**, **75**–**76**, **79**–**81**, **84**, **86**–**87**, **90**–**91**, **95**–**97**, and **105**–**111** having high potency with a mixture of substitution patterns offering total control (Figure 14). These included isoxazoles **68** and **105**, 1,2,5-oxadiazole **91** and a series of seven substituted 2-pyridyl analogues including **4a**, **80**–**81**, **84**, **96**–**97**, and **101** (Figure 14). The *Alternia* fungi appears to be more difficult to effectively target as, while the compounds **4a**, **68**–**69**, **75**–**76**, **79**–**81**, **84**, **87**, **90**–**91**, **95**–**97**, **105,** and **107**–**111** were similar to the inhibitors identified for *Venturiatest,* none reached total control of *Alternia*. The most potent compounds, pyrazole **69**, unsubstituted 2-pyridyl **4a,** and 3-fluoro 2-pyridyl **80** all had 96% control at 250 ppm (Figure 15). The *Botyrtis* fungi was also not completely controlled by the active compounds **4a**, **68**–**69**, **75**–**76**, **81**, **84**, **91**, **95**–**97**, **105**, **107,** and **110**, despite a high level of potency. The SAR around *Botyrtis* was considerably narrower with roughly half the number of earlier analogues reported (Figure 16), despite the higher 500ppm concentration tested. The pyrazole analogue **69** was able to potently inhibit *Botyrtis* infection to 99%, while several other analogues also had potent inhibition (>95%).

Only five 1,2,3-dithiazoles were reported to be active against *Leptosphaeria nodorum* (Figure 17) and the need for an increased concentration of test compound to 1000 ppm, potentially highlighting that *Leptosphaeria nodorum* is more difficult to target. The five compounds reported were all 2-pyridyl substituted **80**, **84**, **87**, **90**, and **96**, but only (*Z*)-4-chloro-*N*-(6-methoxypyridin-2-yl)-5*H*-1,2,3-dithiazol-5-imine (**90**) had total control of the *Leptosphaeria nodorum* infection.

The fungi *Septoria tritici* had a similar profile to *Leptosphaeria nodorum*, with only five potent compounds reported: **84**, **87**, **98**, **109**, and **112** (Figure 18). The most potent four of the five compounds reported were 2-pyridyl substituted **84**, **87**, **98,** and **112**. The 4-methyl 2-pyridyl **87** and 3-methoxy 2-pyridyl **112** were the most potent, with 100% control of *Septoria tritici* infection.

Interestingly, the final set of results of inhibitors against *Pyricularia* revealed only two highly active compounds. These two compounds, (*Z*)-4-chloro-*N*-(6-methoxypyridin-2-yl)-5*H*-1,2,3-dithiazol-5-imine (**90**) and (*Z*)-4-chloro-*N*-(isoxazol-3-yl)-5*H*-1,2,3-dithiazol-5-imine (**68**), were both able to control 100% of the *Pyricularia* infection even at the lower concentration of 250 ppm. The lack of further SAR may (or may not) indicate that, while two highly active compounds are reported this infection was the most difficult to treat.

**Figure 20:**
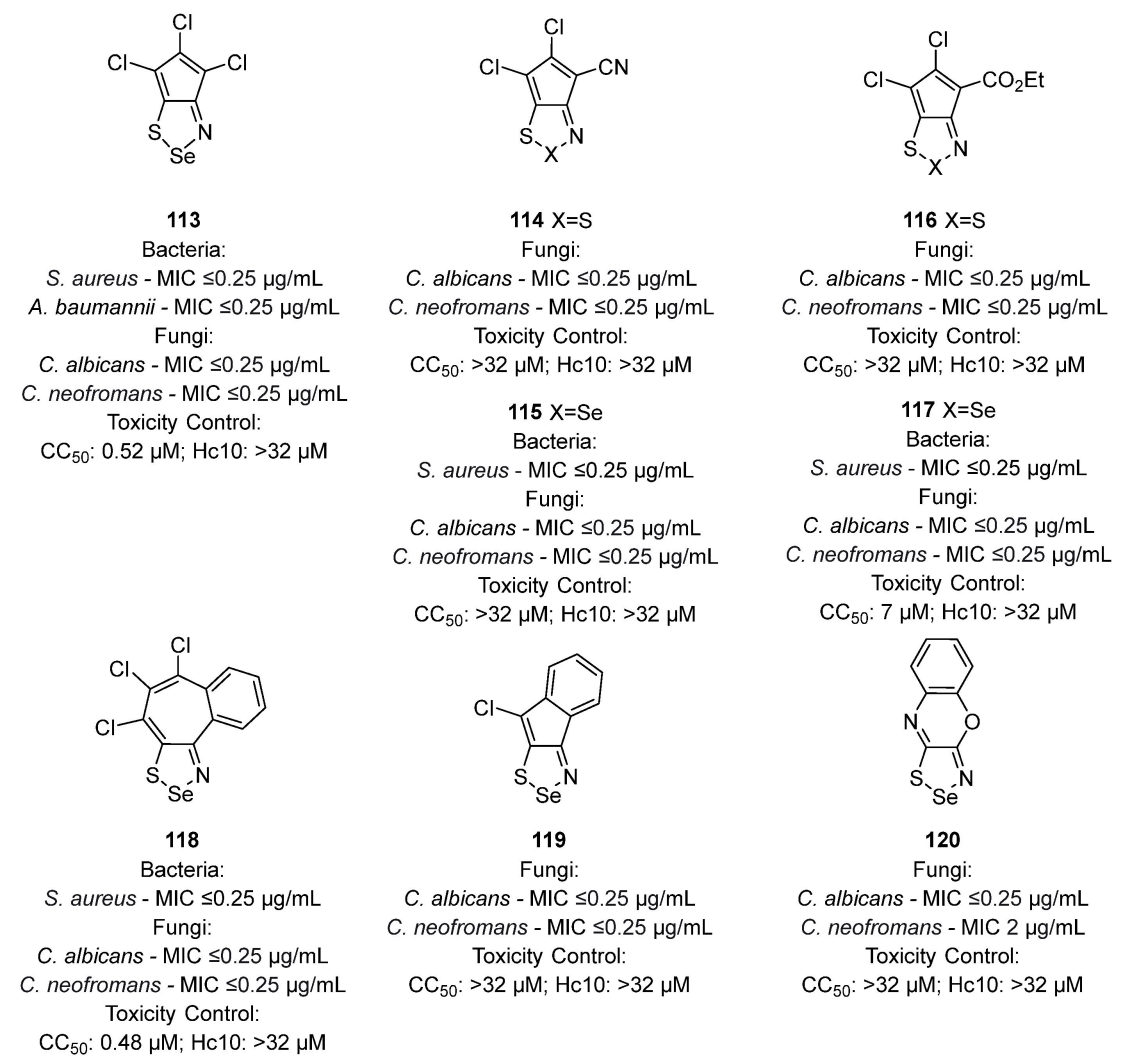
Summary of the most active antifungal 1,2,3-dithiazoles and 1,2,3-thiaselenazoles from the 2020 study by our group [55].

More recently, a 2020 study by our group reported a set of 1,2,3-dithiazoles and matched pair 1,2,3-thiaselenazoles as antimicrobials [55]. The rare 1,2,3-thiaselenazoles were synthesized by sulfur extrusion and selenium insertion into 1,2,3-dithiazoles [55,56]. This work was part of the Community for Antimicrobial Drug Discovery (CO-ADD) project to develop new lead compounds for priority targets with an unmet clinical need [57]. The compounds were screened against *S. aureus*, *A. baumannii*, *C. albicans,* and *C. neoformans var. grubii.* with a toxicity counter screen in HEK293 cells and an additional hemolysis assay (Hc10) (Figure 20). These strains are considered by the World Health Organization (WHO) to be the highest priority to develop novel antibiotics for control of these bacteria and fungi [58].

The compounds **113**–**120** demonstrated potency against several of the strains tested, with the 1,2,3-thiaselenazoles tending to be more active (Figure 20). The 4,5,6-trichlorocyclopenta[*d*][1,2,3]thiaselenazole (**113**) demonstrated potent activity against Gram-positive bacteria *S. aureus* (MIC = ≤0.25 μg/mL), Gram-negative bacteria *A. baumannii* (MIC = ≤0.25 μg/mL) along with antifungal activity against *C. albicans* and *C. neofromans* (both MIC ≤0.25 μg/mL). The trichoro analogue **113** had some toxicity (CC_50_ = 0.52 μM), whereas both the 4-cyano **114**/**115** and 4-ethylester **116**/**117** 1,2,3-dithiazole/1,2,3-thiaselenazole matched pair analogues showed limited to no toxicity (all CC_50_ = >32 μM, apart from **117** = CC_50_ = 7 μM). The 4-cyano analogues **114**/**115** were both active against *C. albicans* and *C. neofromans* (both MIC ≤0.25 μg/mL); however, the 1,2,3-thiaselenazole also had activity against *S. aureus* (MIC = ≤0.25 μg/mL). This activity trend was matched exactly by the 4-ethylester analogues **116**/**117**. The 4,5,6-trichlorobenzo[6,7]cyclohepta [1,2-*d*][1,2,3]thiaselenazole (**118**) analogue matched the profile of **115** and **117** albeit with some toxicity (CC_50_ = 0.48 μM). Interestingly, the activity profiles of 8-chloroindeno[1,2-*d*][1,2,3]thiaselenazole (**119**) and benzo[*b*][1,2,3]thiaselenazolo[5,4-*e*][1,4]oxazine (**120**) were similar with antifungal activity against *C. albicans* and *C. neofromans* (all MIC = ≤0.25 μg/mL, apart from **120**, *C. neofromans* = 2 μg/mL). Taken together these results demonstrate an ability for the 1,2,3-dithiazole/1,2,3-thiaselenazole to inhibit a broad range of challenging and clinically relevant bacteria and fungi [55,58].

### 3.2. Antiviral Activities of 1,2,3-Dithiazoles

**Figure 21:**
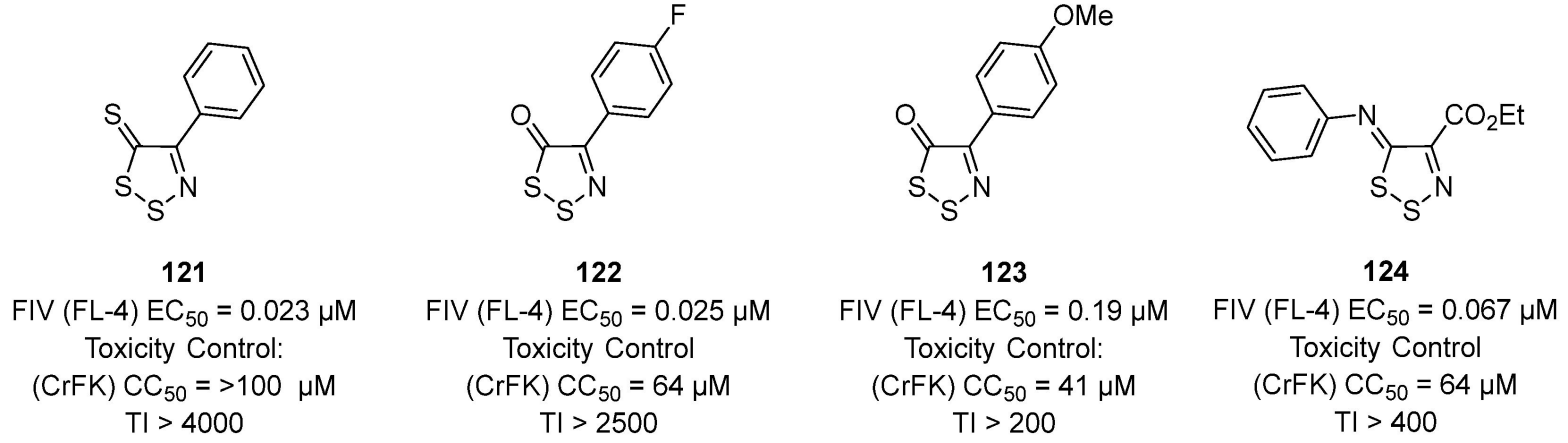
Summary of the initial report of antiviral activity of the 1,2,3-dithiazole scaffold.

The first antiviral activities on the 1,2,3-dithazole scaffold were reported in 2016 by Hilton et al. [17]. A series of 5-thieno-, 5-oxo-, and 5-imino-1,2,3-dithiazole derivatives were screened against Feline Immunodeficiency Virus (FIV) as a model for HIV infection. The rationale of using the 1,2,3-dithiazoles to target the nucleocapsid protein was that it could potentially act as a zinc ejector by utilizing the disulfide bridge [59,60,61,62]. The compounds were tested for antiviral effects in a feline lymphoid cell line (FL-4) and tested for toxicity using Crandell-Rees feline kidney (CrFK) cells (Figure 21). The four highlighted compounds, **121–124**, were the most potent antivirals with the largest toxicity window (ratio of FL-4/CrFK). The 4-phenyl-5*H*-1,2,3-dithiazole-5-thione (**121**) had an excellent ratio of activity vs. toxicity (>4000) and potency of EC_50_ = 23 nM. The 4-(4-fluorophenyl)-5*H*-1,2,3-dithiazol-5-one (**122**) was equipotent to **121** with a small amount of toxicity at higher concentrations (CC_50_ = 64 μM). The 4-methoxy analogue **123** had a drop of almost 8-fold in potency, with the ethyl (Z)-5-(phenylimino)-5*H*-1,2,3-dithiazole-4-carboxylate (**124**) analogue had an almost 3-fold drop, both with a comparable toxicity profile.

**Figure 22:**
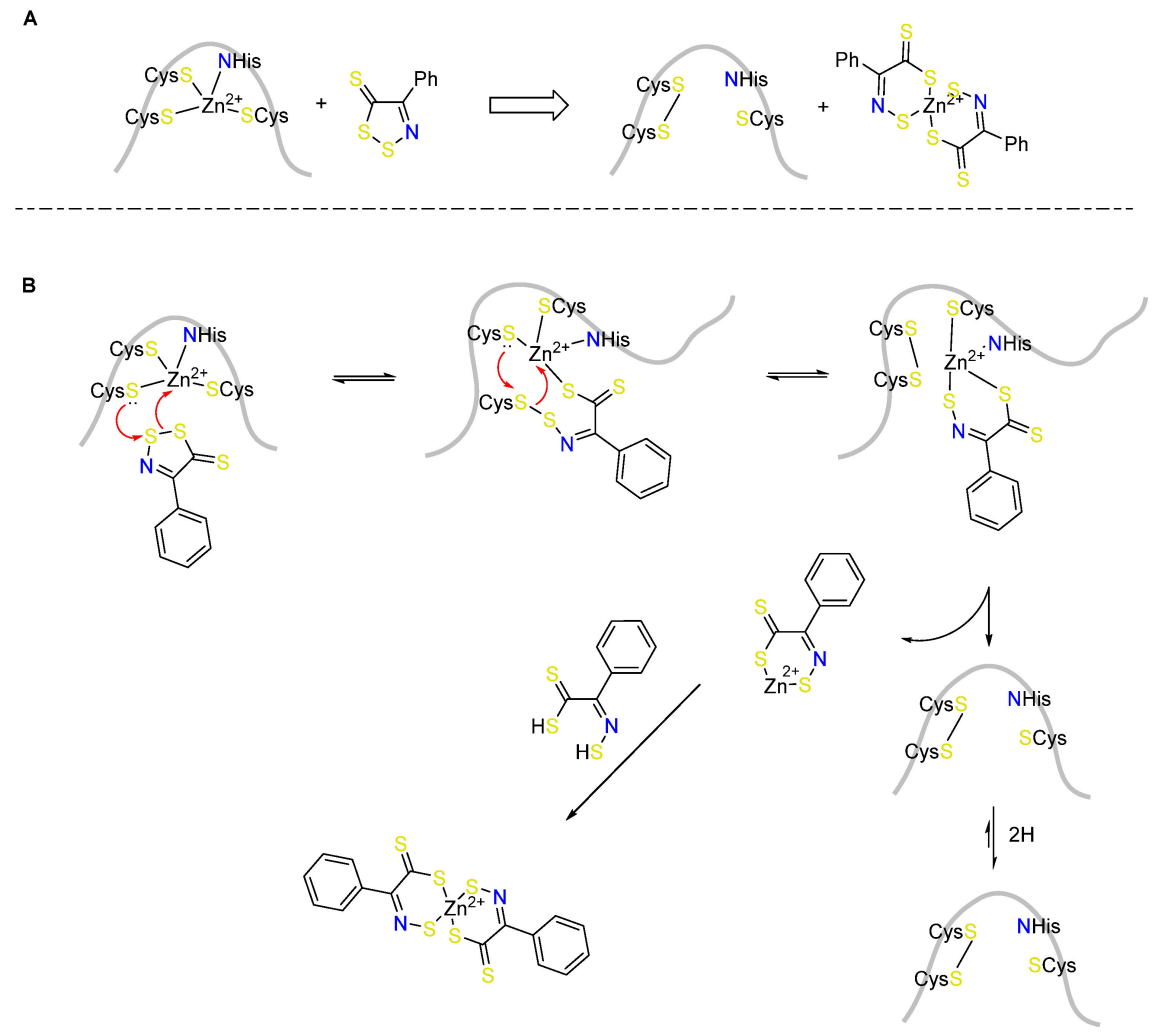
Proposed redox mechanism for 1,2,3-dithiazoles mediated zinc ejection of the FIV nucleocapsid protein. (**A**) Summary of reaction; (**B**) Detailed reaction pathway analysis.

**Figure 23:**
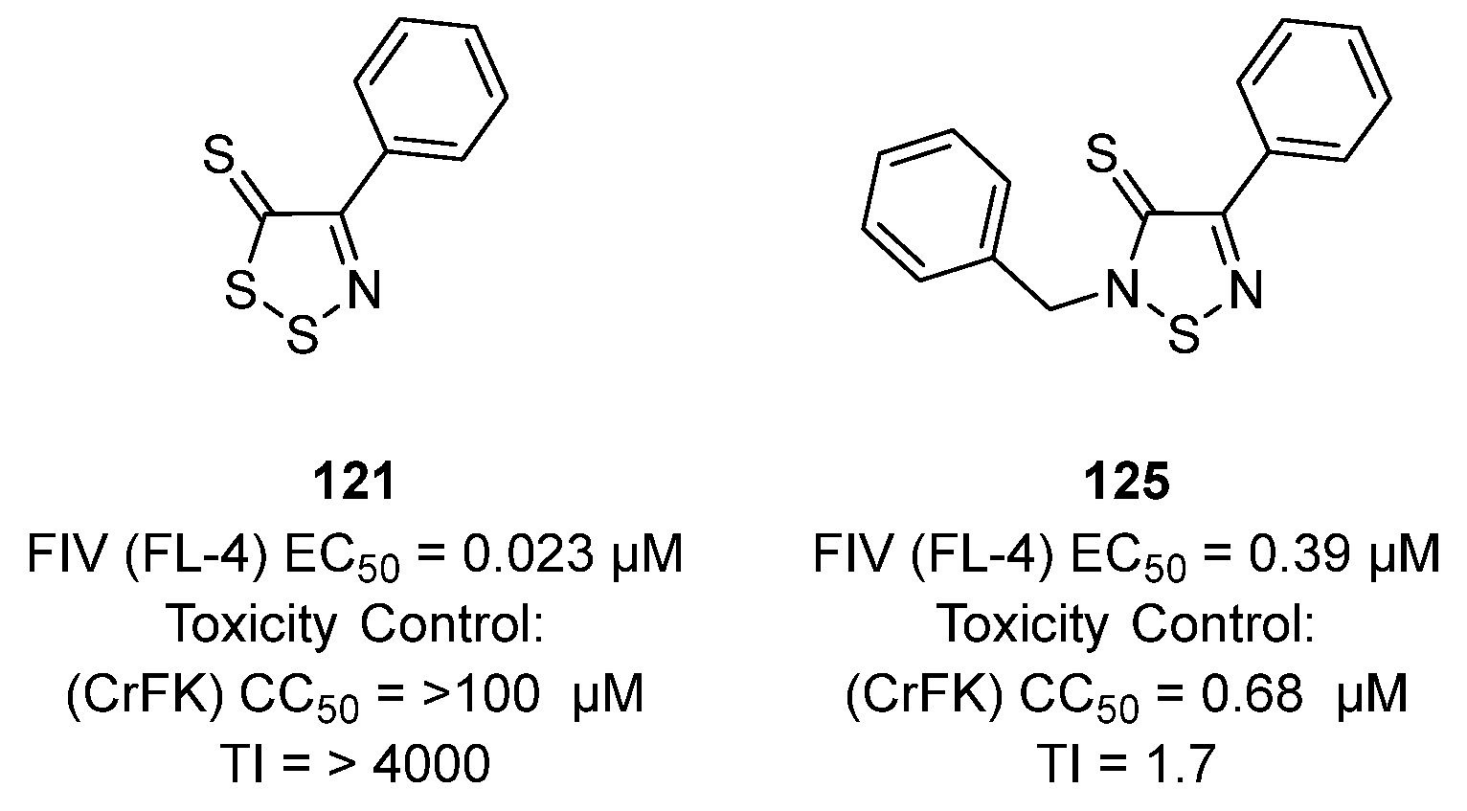
Direct comparison of **121** against **125** with the disrupted 1,2,5-thiadiazole-3(2*H*)-thione ring system.

The proposed mechanism of action was modelled on previously experimental reports (Figure 22) [59,60,61,62]. Zinc ejection from nucleocapsid protein starts with Zn^2+^ coordinated to cysteine thiol(ate)s reacting with the disulfide of the 1,2,3-dithiazole core to generate a transient intermediate disulfide. This complex then rearranges to form an intramolecular protein disulfide, which has a consequent reduction in zinc ion affinity. This results in the zinc being ejected from the protein in a similar mechanism as previously reported for the HIF1alpha/P300 interaction triple zinc finger [59]. To indirectly prove the mechanism in addition to computational modelling, a disrupted disulfide bridge of analogue **121**, compound **125** was synthesized (Figure 23) [47], demonstrating that the disulfide was required for activity.

**Figure 24:**
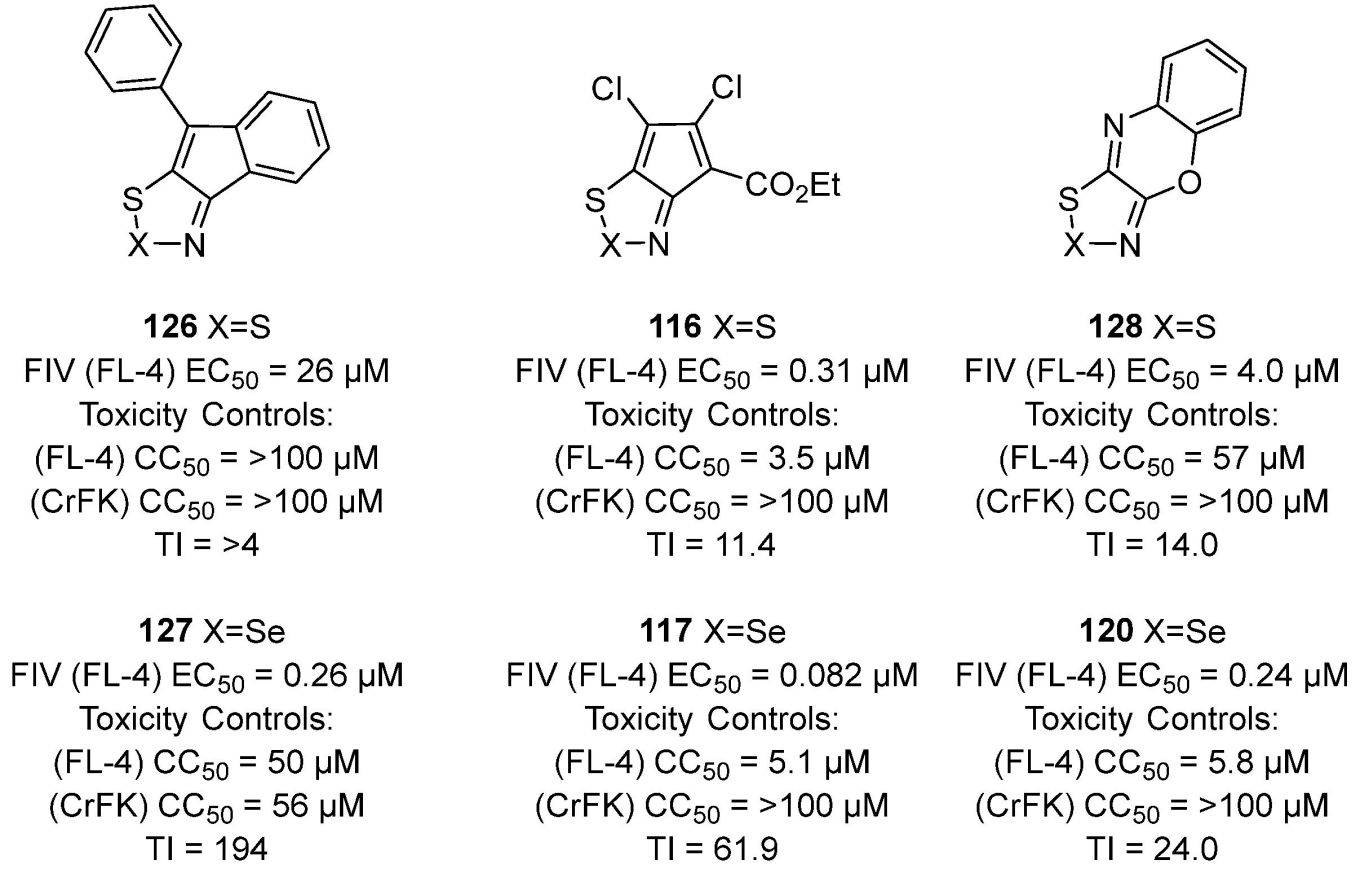
Summary active antiviral matched pair 1,2,3-dithiazoles and 1,2,3-thiaselenazoles.

This idea was followed up in 2019 by our group [63], investigating the same inhibitors later reported as antimicrobials [55]. The key rationale behind this subsequent work was the further investigation of the disulfide bridge involvement on antiviral efficacy with a matched pair side by side comparison between the 1,2,3-dithiazoles and the 1,2,3-thiaselenazole scaffold. Where the weaker S-Se vs. S-S bond should assist in increasing the antiviral efficacy. This followed on from a previous report of a successful selenide isosteric replacement to several literature nucleocapsid protein inhibitors, including DIBA-4 to DISeBA-4 HIV inhibitors, resulting in good potency and only a very limited associated toxicity [64]. The antiviral efficacy of the 1,2,3-dithiazole scaffold was tested using FL-4 cells, but in this study an additional toxicity assay was preformed directly on the FL-4 cells (Figure 24). The 8-phenylindeno[1,2-*d*][1,2,3]dithiazole (**126**) was only weakly active, while the selenium analogue **127** demonstrated a 10-fold boost in potency to EC_50_ = 0.26 μM with only limited toxicity. The ethyl 5,6-dichlorocyclopenta[*d*][1,2,3]dithiazole-4-carboxylate (**116**) had a similar profile with an EC_50_ = 0.26 μM, while the selenium analogue **117** was almost 4-fold more potent. The difference between the benzo[*b*][1,2,3]dithiazolo[5,4-*e*][1,4]oxazine (**128**) and selenium analogue **120** was even more pronounced with an almost 17-fold increase in potency. These results highlight the advantages of including selenium in the 1,2,3-dithiazole scaffold.

**Figure 25:**
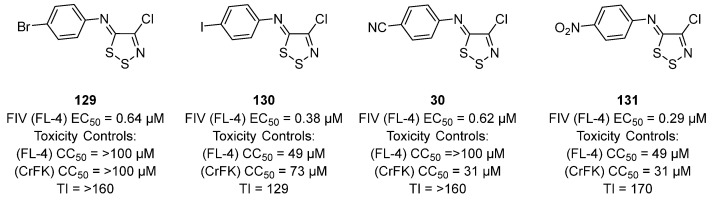
Initial hit compounds from the 1,2,3-dithiazole library.

**Figure 26:**
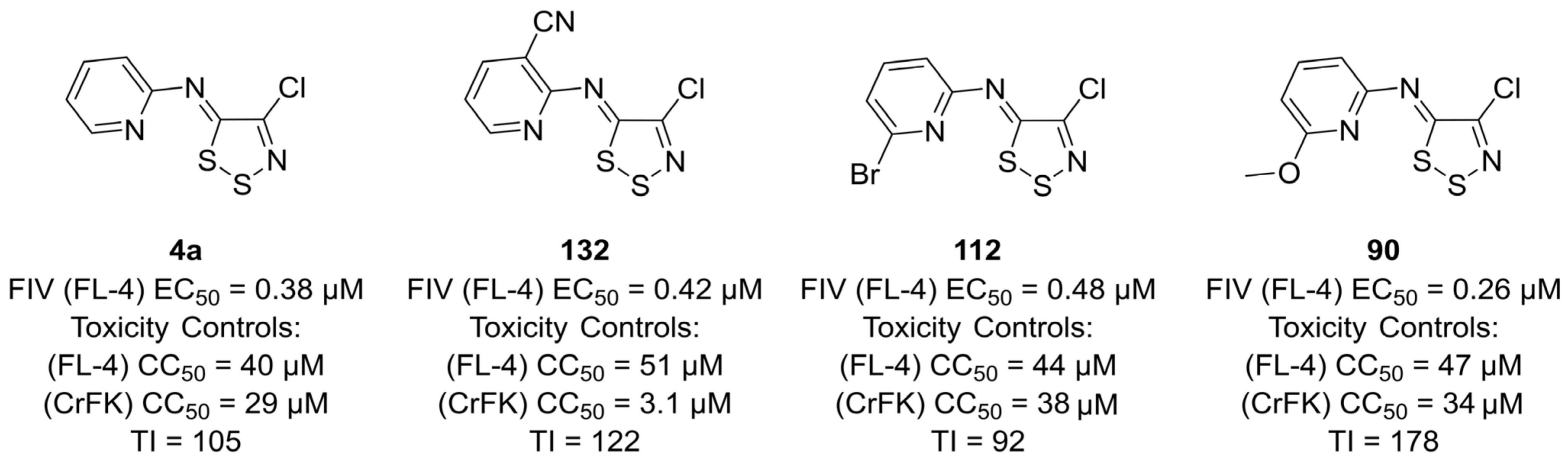
2-Pyridyl substituted 1,2,3-dithiazoles active against FIV.

**Figure 27:**
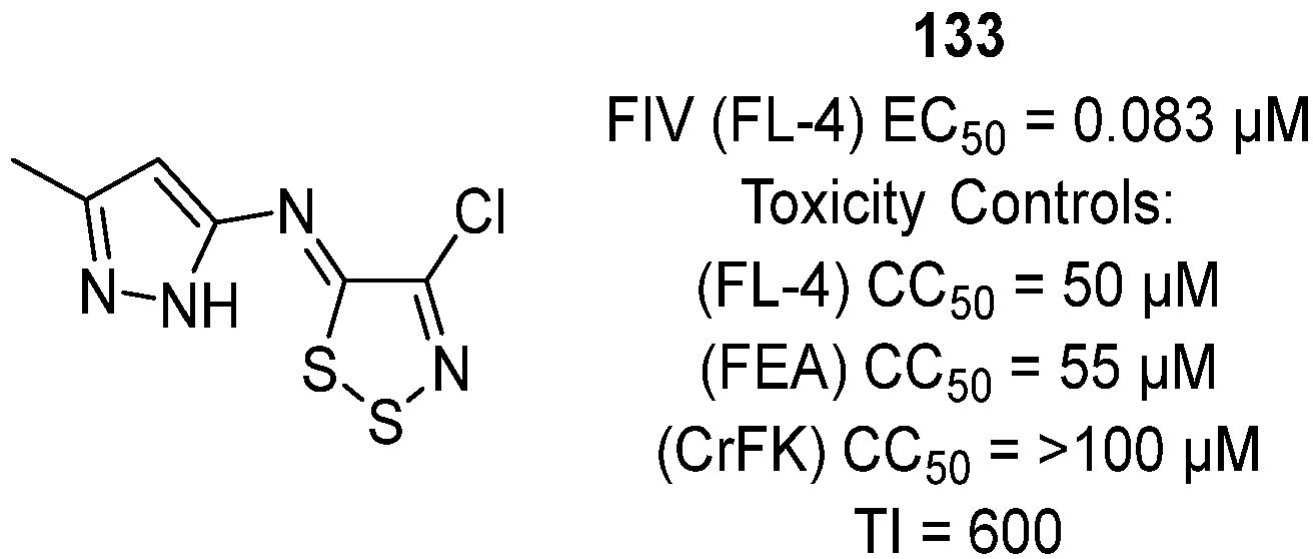
Most potent compound **133** active agiants FIV in the 2022 study by our group [36].

More recently, a further extension of investigation of the 1,2,3-dithiazoles in 2022 by our group evaluated a further series of 1,2,3-dithiazoles against FIV as a model for HIV infection [36]. The rationale of this investigation was to find a tractable series of 1,2,3-dithiazoles with consistently high potency and lower toxicity to further advance the scaffold. The antiviral screening was performed using FL-4 cells, with a direct toxicity assay on FL-4 cells in addition to CrFK and feline embryo cell line (FEA) cells (Figure 25, Figure 26 and Figure 27).

The initial hit compounds from the 1,2,3-dithiazole library yielded a series of 4-position substituted phenyl analogues **30** and **129–131** with a range of activities EC_50_ = 0.26–0.48 μM and limited toxicity (Figure 25). Another trend observed within the series was activity across a number of 2-pyridyl substituted analogues **4a**, **90**, **112** and **132**, at a similar level to the earlier analogues but with a divergent SAR profile (Figure 26). The most promising compound identified in this work was the pyrazole (*Z*)-4-chloro-*N*-(3-methyl-1*H*-pyrazol-5-yl)-5*H*-1,2,3-dithiazol-5-imine (**133**) that showed good antiviral potency EC_50_ = 0.083 μM with very limited toxicity (Figure 27).

**Figure 28:**
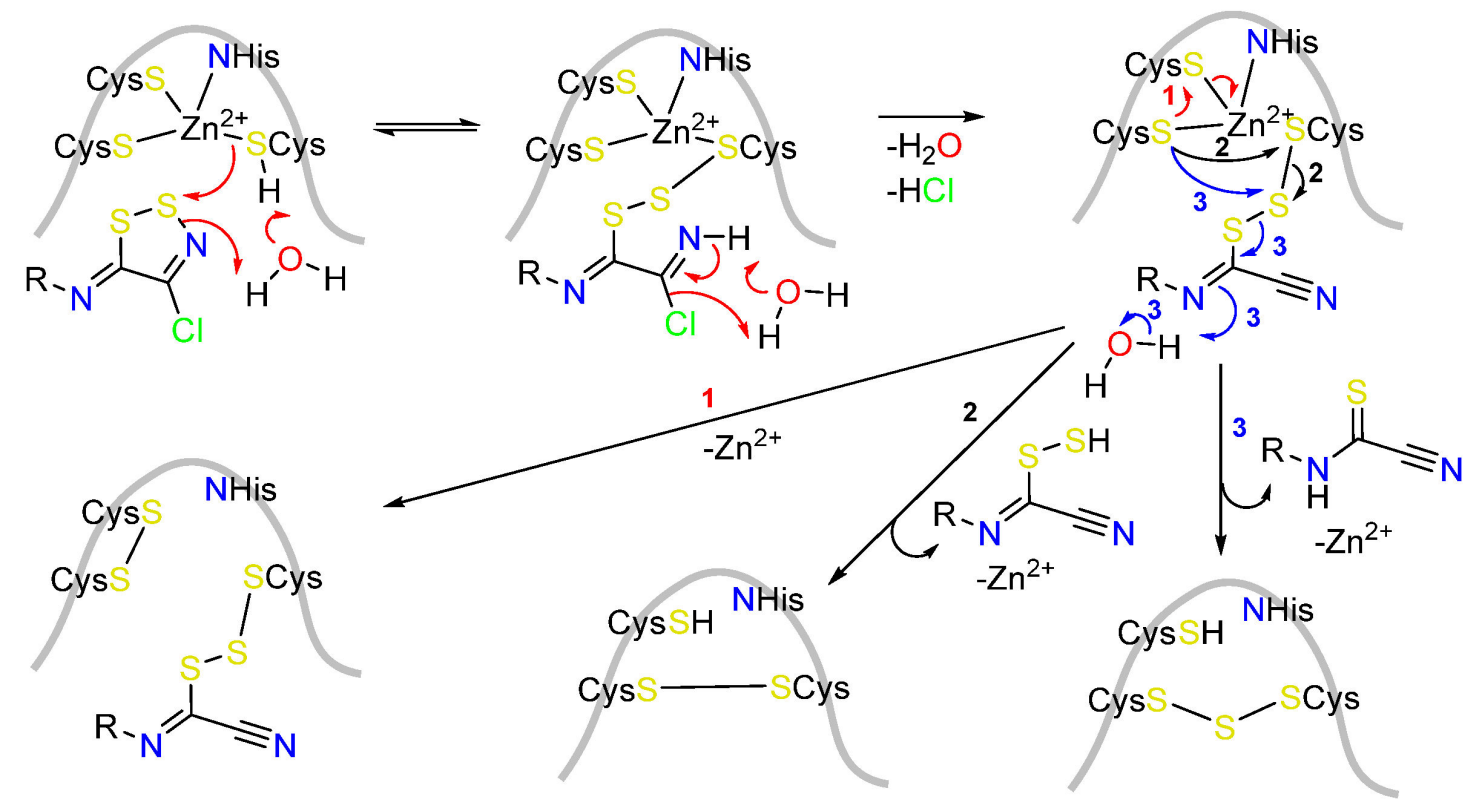
Proposed mechanism of action of the 4-choro-1,2,3-dithiazole series.

The proposed mechanism of action on the nucleocapsid protein of this 4-chloro-1,2,3-dithiazol-5-imine series is different to the C4 substituted version previously reported (Figure 22). The DFT calculations and previous ANRORC-style rearrangement reported on this scaffold suggest that there will be a ring opening and chloride elimination. An outline mechanism would be a Zn^2+^-coordinating cysteine thiol(ate) reacts with 2-S of the 1,2,3-dithiazole core mediated by water to generate a transient trisulfide. This is then followed by a rearrangement to a more thermodynamically stable cyano functionality, resulting in the loss of HCl and water from the system. The, disulfide then rearranges to form an intramolecular protein disulfide with consequent reduction in zinc ion affinity. The zinc ion is then ejected to form a stable complex, with or without adducts (Figure 28). In addition to the literature rearrangement examples [38,39,40], we also provided extensive computational modelling to support the mechanistic rationale provided.

### 3.3. Anticancer Activities of 1,2,3-Dithiazoles

**Figure 29:**
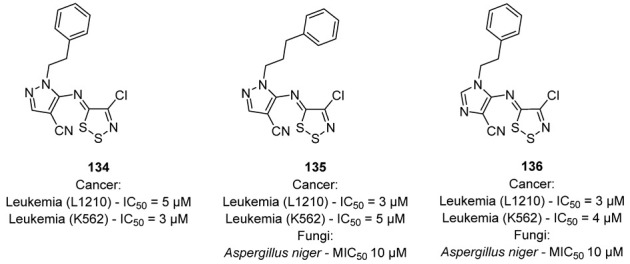
Initial 1,2,3-dithiazoles reported with anticancer activity in 2002 by Baraldi et al. [16].

Initial reports of anticancer activity with the 1,2,3-dithiazole scaffold were reported in 2002 by Baraldi et al. [16]. A set of ten 1,2,3-dithiazoles were prepared and screened across multiple antimicrobial and anticancer therapeutic targets. Several of the compounds **134**–**136** showed low signal digit micromolar potency against the leukemia cell lines L1210 and K562 (Figure 29). While the overall SAR within the series was flat, this first phenotypic report showed tractable activity across both cell lines. The antibacterial screen showed limited activity, but the antifungal screening identified **135** and **136** as having some activity against *Aspergillus niger* at MIC_50_ = 10 μM. This further supports the overall tractability of this scaffold as an antifungal.

**Figure 30:**
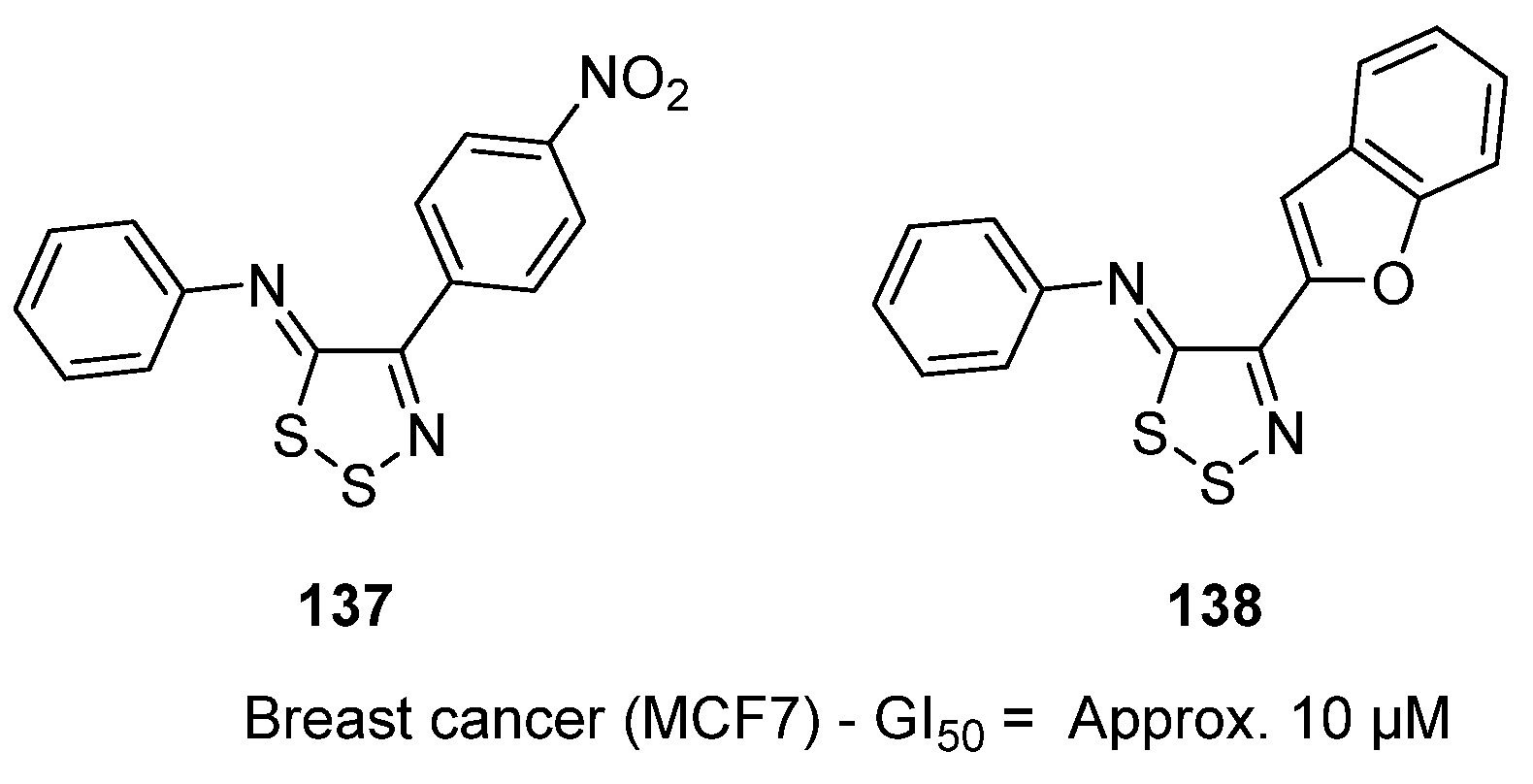
Substituted C4 1,2,3-dithiazoles with activity against breast cancer.

The earlier 2009 study reported in Section 3.1 by Rakitin et al. also screened the series of C-4 substituted dithiazoles against two breast cancer cell lines, MCF7 and MDA-MB-231 [13]. Limited activity was observed on the MDA-MB-231 cell line across the series. However, (*Z*)-4-(4-nitrophenyl)-*N*-phenyl-5*H*-1,2,3-dithiazol-5-imine (**137**) and the benzofuran-2-yl analogue **138** showed 50% growth inhibition after 72 h at approximately 10 μM (Figure 30). These results lay the groundwork to expand the chemical space to further investigate the anticancer 1,2,3-dithiazole SAR.

**Figure 31:**
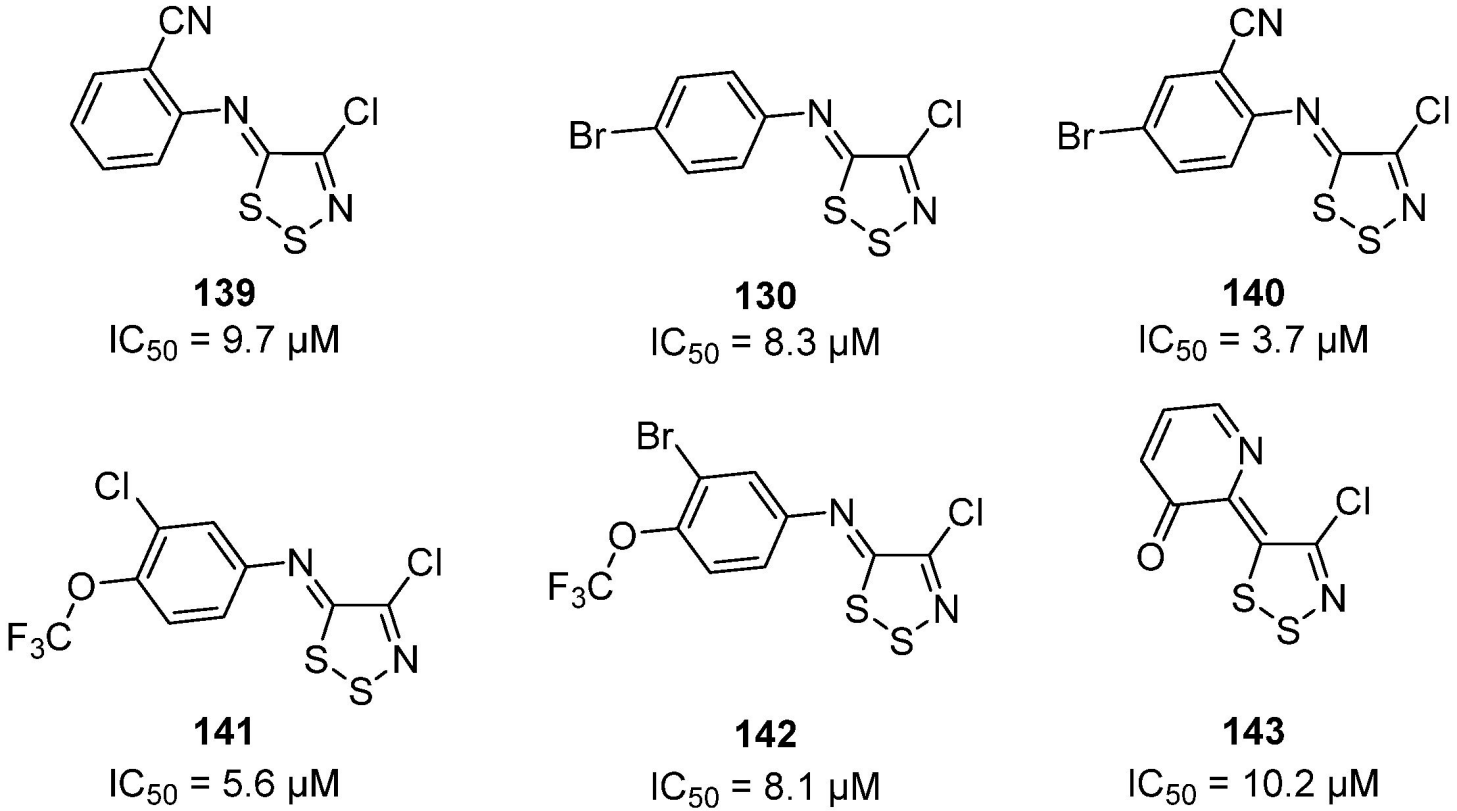
Anticancer 1,2,3-dithiazoles ASCT2 transport inhibitors.

Subsequent to these phenotypic reports of 1,2,3-dithiazoles as anticancer compounds in various cell lines, a just over forty compound 1,2,3-dithiazole library was screened by Indiveri et al. against a transporter target over-expressed in various cancers, the glutamine-amino acid transporter ASCT2 in 2012 [65]. Interactions with scaffold proteins and post-translational modifications regulate the stability, trafficking, and transport activity of ASCT2 [66]. The expression of ASCT2 has been shown to increase in cells with rapid proliferation, including stem cells and inflammation, this enables delivery of the increased glutamine requirements [67]. This same mechanism can be hijacked by cancer promoting pathways to fulfill glutamine demand and facilitate rapid growth by over-expression of ASCT2 [68]. In addition to being described as an anticancer target, ASCT2 also has the ability to traffic virions to infect human cells [69]. A series of 1,2,3-dithiazoles were synthesized and evaluated as transporter inhibitors. While many compounds were in-active at 30 μM, six compounds showed activity at IC_50_ = ~10 μM or below (Figure 31). These compounds potently inhibited the glutamine/glutamine transport catalyzed by ASCT2.

**Figure 32:**
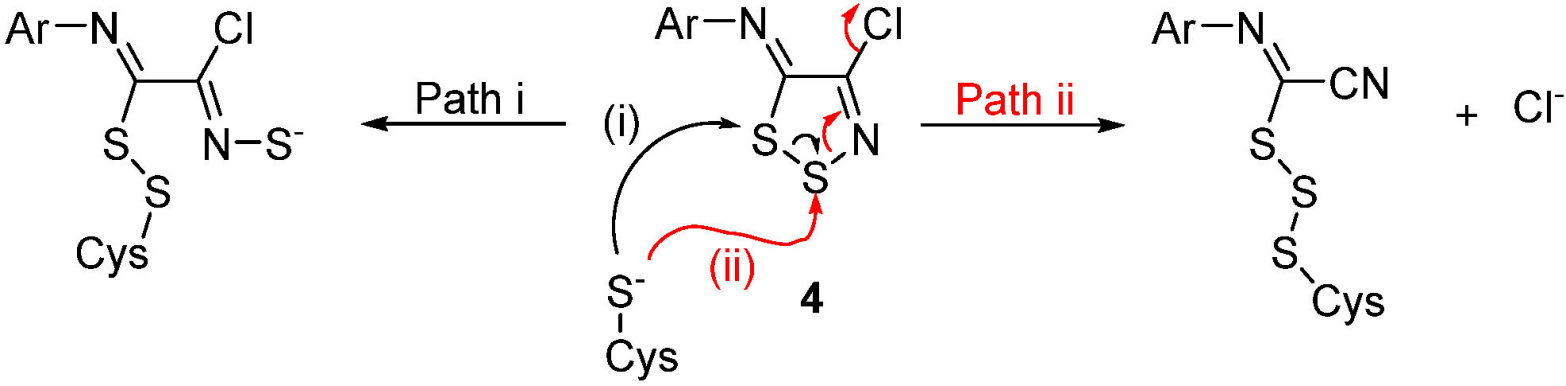
Two mechanisms are proposed, with nucleophilic attack at S2 to likely be preferred.

The inhibition was shown to be non-competitive. The inhibition was also reversed by addition of dithiothreitol (DTE), indicating the reaction with protein Cys formed adducts, indicating that the reaction was likely going *via* an ANRORC-style rearrangement. Modelling, including molecular and quantum mechanical studies (MM and QM, respectively) and Frontier Orbital Theory (FOT) on 1,2,3-dithiazole models showed pathway (ii) was more likely, which is also supported by previous reports on the 1,2,3-dithiazole (Figure 32) [38,39,40].

**Figure 33:**
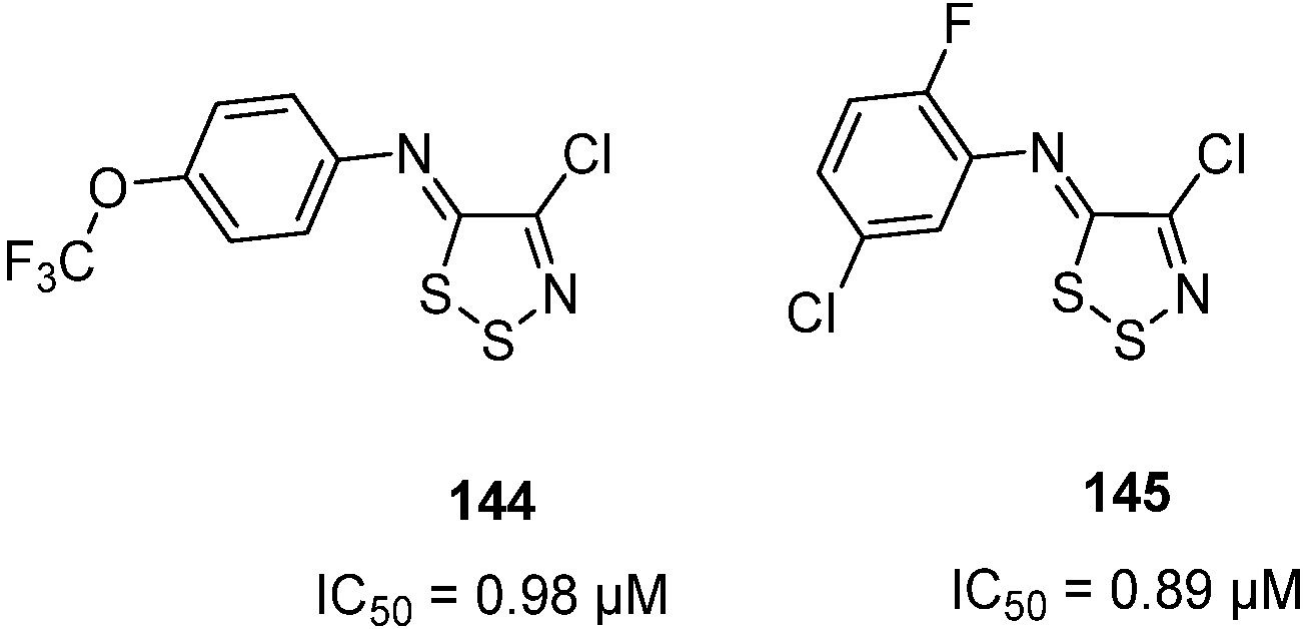
Anticancer 1,2,3-dithiazoles targeting transporter protein LAT1 (SLC7A5).

The ASCT2 report was followed up by a screening of just over fifty 1,2,3-dithiazoles by Indiveri et al. against the LAT1 transporter in 2017 [70]. ASCT2 and LAT1 are both amino acid transporters that are overexpressed in cancer [71]. Subsequently, a number of inhibitors have been reported against both ASCT2 and LAT1, with one LAT1 inhibitor JPH203 used in a recent phase 1 clinical trial [72]. The results of the library screen were eight compounds with inhibition of >90% at 100 µM. The two most potent compounds were **144** and **145** with an IC_50_ = <1 µM (Figure 33).

The inhibition kinetics, performed on the two best inhibitors (**144** and **145**), indicated a mixed type of inhibition with respect to the substrate. The inhibition of LAT1 was still present after removal of the compounds from the reaction mixture, indicating irreversible binding. However, this effect could be reversed by the addition of dithioerythritol, a S-S reducing agent, which supports the rationale of the formation of disulfide(s) bonds between the compounds and LAT1. Molecular modelling of **144** and **145** on a homology model of LAT1, highlighted the interaction with the substrate binding site and the formation of a covalent bond with the residue C407. This was further supported by a more detailed study reported in 2021 by Marino et al., which also highlighted the need for a molecule of water in the reactive pathway [73].

**Figure 34:**
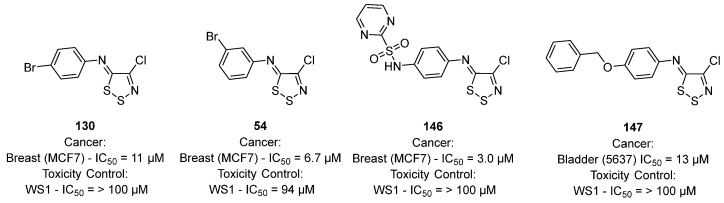
Initial screening highlighted results from the 2021 cancer panel by our group [74].

**Figure 35:**
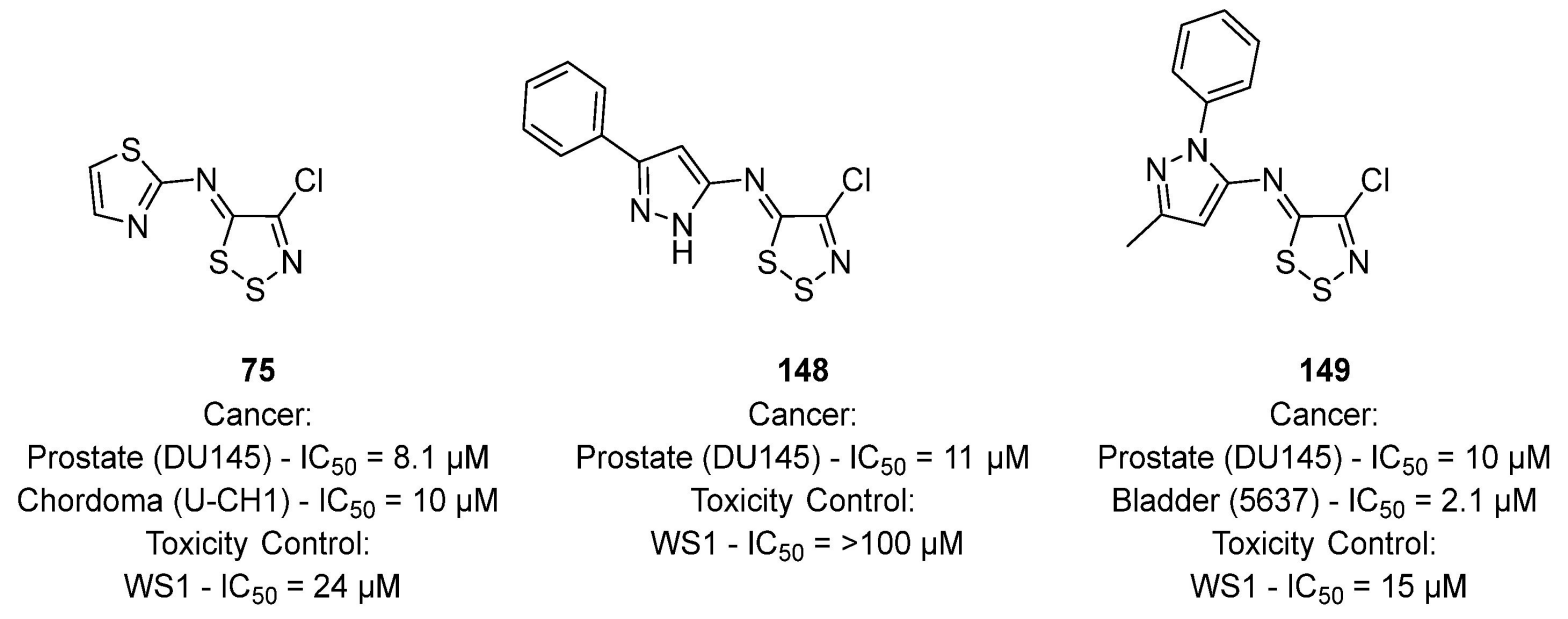
5-membered heteroatomic analogues highlighted results from the 2021 cancer panel by our group [74].

**Figure 36:**
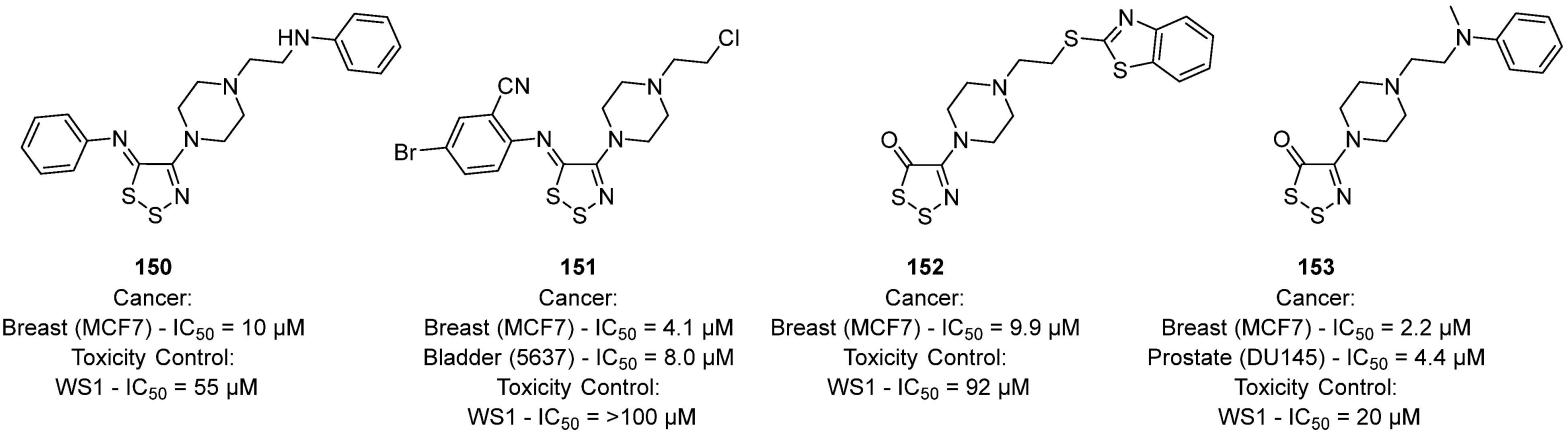
C4 substituted 1,2,3-dithazole analogues highlighted results from the 2021 cancer panel by our group [74].

More recently, an extension of the phenotypic reports of 1,2,3-dithiazoles as anticancer agents was published in 2021 by our group [74]. A library of just under forty 1,2,3-dithiazole analogues were screened on a series of cancer cell lines including breast, bladder, prostate, pancreatic, chordoma and lung; with a skin fibroblast cell line as a non-specific toxicity control (Figure 34, Figure 35 and Figure 36).

Initial results were encouraging (Figure 34) with (*Z*)-4-(4-bromophenyl)-*N*-phenyl-5*H*-1,2,3-dithiazol-5-imine (**130**) and the corresponding 3-position bromo analogue **54** demonstrated potency against breast cancer cell line MCF7 (IC_50_ = 11 and 6.7 μM, respectfully). This was followed by the identification of (*Z*)-*N*-(4-((4-chloro-5*H*-1,2,3-dithiazol-5-ylidene)amino)phenyl)pyrimidine-2-sulfonamide (**146**) with good activity against breast cancer (MCF7—IC_50_ = 3.0 μM) and no observed toxicity (WS1—IC_50_ = >100 μM). Interestingly, the (*Z*)-*N*-(4-(benzyloxy)phenyl)-4-chloro-5*H*-1,2,3-dithiazol-5-imine (**147**) analogue showed a preference for bladder cancer inhibition (5637—IC_50_ = 13 μM) with no observed toxicity (WS1—IC_50_ = >100 μM).

This was followed by screening a small focused 5-membered heteroatomic compounds, which identified a trend of potency against prostate cancer (Figure 35). (*Z*)-4-chloro-*N*-(thiazol-2-yl)-5*H*-1,2,3-dithiazol-5-imine (**75**) and the two pyrazoles (**148** and **149**) all showed activity against prostate cancer (DU145—IC_50_ = 8–11 μM). The thiazole analogue **75** also showed activity against the chordoma cell line (U-CH1—IC_50_ = 10 μM), albeit with some limited toxicity (WS1—IC_50_ = 24 μM). The (*Z*)-4-chloro-*N*-(3-methyl-1-phenyl-1*H*-pyrazol-5-yl)-5*H*-1,2,3-dithiazol-5-imine (**149**) also showed low single digit micromolar activity against bladder cancer (5637—IC_50_ = 2.1 μM); unfortunately, this was coupled with some associated toxicity (WS1—IC_50_ = 15 μM).

Subsequent to this screening, a series of C-4 substituted analogues were evaluated resulting in a trend of activity against breast cancer (MCF7) (Figure 36) [14]. The most potent compounds **150–153** (IC_50_ = 2–10 μM) did not show any defining SAR characteristics. In addition to this activity, (*Z*)-5-bromo-2-((4-(4-(2-chloroethyl)piperazin-1-yl)-5*H*-1,2,3-dithiazol-5-ylidene)amino)benzonitrile (**151**) demonstrated good potency against bladder cancer (5637—IC_50_ = 8.0 μM), while 4-(4-(2-(methyl(phenyl)amino)ethyl)piperazin-1-yl)-5*H*-1,2,3-dithiazol-5-one (**153**) was potent against prostate cancer (DU145—IC_50_ = 4.4 μM), albeit with some observed toxicity (WS1—IC_50_ = 20 μM) in the case of **153**. Interestingly, the 4-chloro-1,2,3-dithiazole of the earlier analogues was not required for activity suggesting there may be multiple mechanisms of action.

### 3.4. Other Biological Applications

#### 3.4.1. Melanin Synthesis Inhibitors

**Figure 37:**
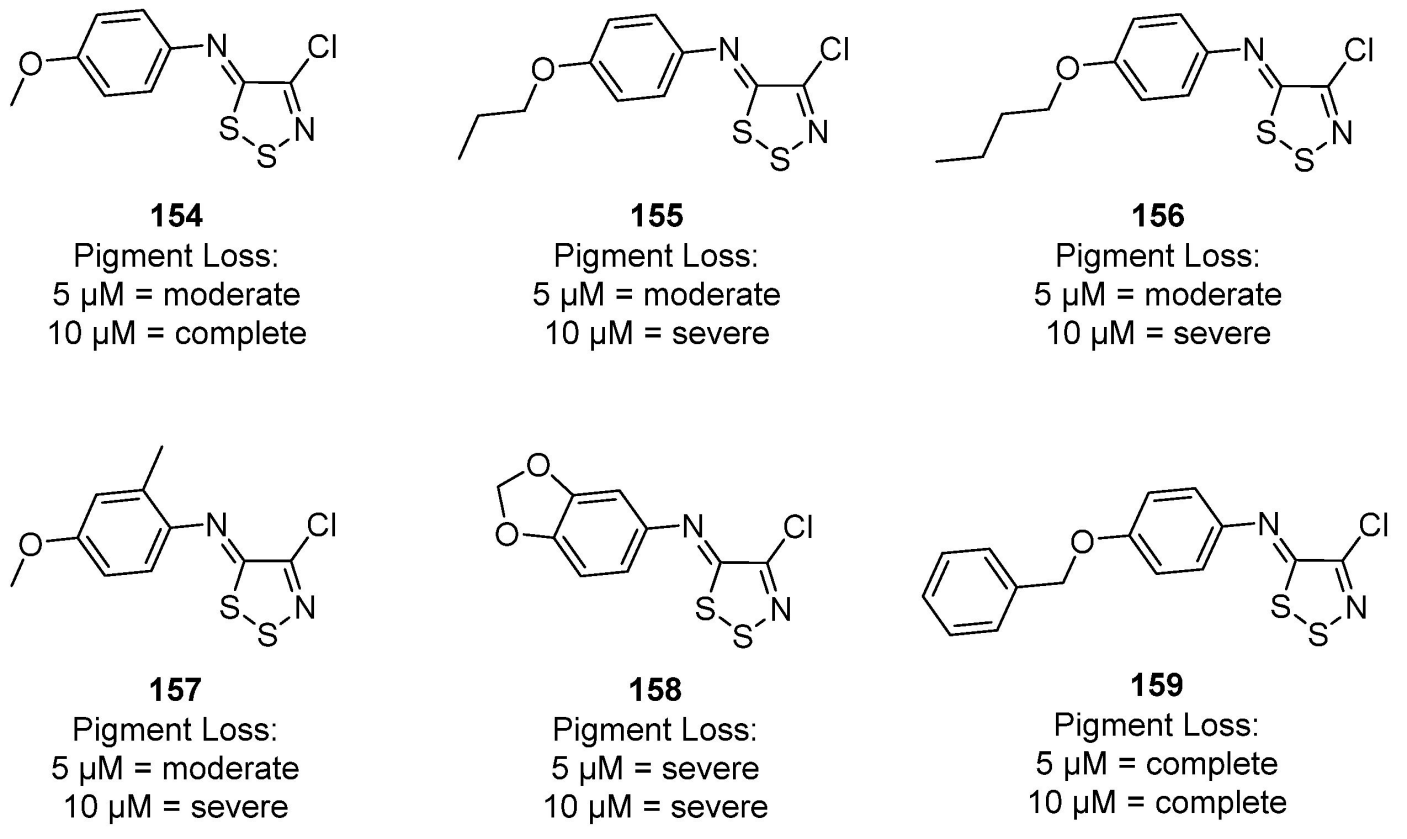
1,2,3-dithiazoles demonstrating In vivo pigment loss in *Xenopus laevis* embryos.

In 2015, a phenotypic screen was carried out using *Xenopus laevis* embryos by Skourides et al. [19]. This led to the identification of a series of 1,2,3-dithiazoles, which caused loss of pigmentation in melanophores and the retinal pigment epithelium (RPE) of developing embryos (Figure 37). This effect was independent of the developmental stage of initial exposure and was reversible. While the target was not elucidated, SAR of the series indicated that the presence of the mesmerically electron-donating methoxy group was important for pigment loss. Compounds with inductive and/or mesmerically electron-withdrawing groups had no effect on pigment loss.

The (*Z*)-4-chloro-*N*-(4-methoxyphenyl)-5*H*-1,2,3-dithiazol-5-imine (**154**) analogue demonstrated complete pigment loss at 10 μM and moderate at 5 μM. Extension of the methoxy to propyloxy **155** or butyloxy **156** reduced the potency, with the addition of a methyl group in the 2-position had the same effect. The formation of a 3,4-fused methyl catacol **157** increased potency, but did not match the activity of the 4-position methoxy **154**. The extension to form that benzyloxy analogue **158** did boost the potency of **154**, resulting in **158** having complete pigment loss at 5 μM.

**Figure 38:**
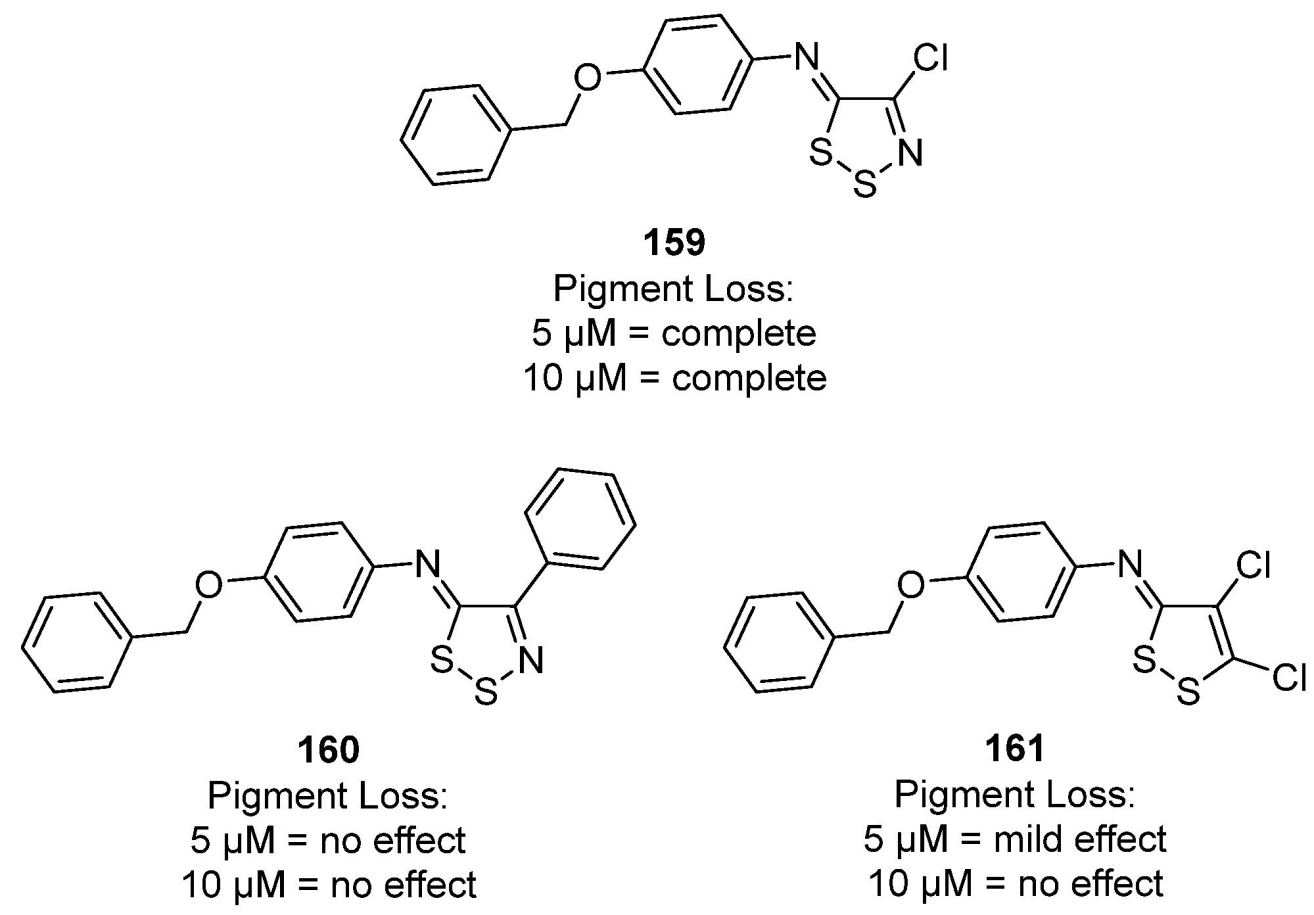
Direct comparison of 1,2,3-dithiazole **159** with disrupted analogues **160** and **161** in In vivo pigment loss in *Xenopus laevis* embryos.

Skourides et al. extensively investigated the structural features driving the phenotypic effects observed with **159**. An analogue of **159** was synthesized in two steps via the oxime route (Scheme 8) [12,13], where the 4-chlorine substituent was replaced with a phenyl group to give compound **160** (Figure 38). A second analogue of **159** was furnished where the nitrogen of the 1,2,3-dithiazole was replaced with a chlorocarbon in one step from Boberg salt [75,76] to give compound **161** (Figure 38) [75,77].

The replacement of the 4-chlorine substituent yielded compound **160**, which showed no phenotypic affect. This supports the idea of an ANRORC-style ring opening mechanism, as the chlorine is a good nucleofuge that facilities the ring opening mechanism [40]. The second analogue **161**, showed some mild activity at 5 μM, while at 10 μM, mild toxicity and developmental defects were observed. This again pointed towards a ring opening ARONOC style mechanism, but more work needs to be done to establish the exact mechanism of action [19].

#### 3.4.2. Antifibrotic Collagen Specific Chaperone hsp47 Inhibitor

Other activities of 1,2,3-dithiazoles include hit compound methyl 6-chloro-3*H*-benzo[*d*][1,2,3]dithiazole-4-carboxylate 2-oxide (**162**), which was reported twice, once in 2005 [18] and the second in 2010 [19]. These reports were both high-throughput screens of the compound library, one from Maybridge Chemical Co., Cornwall, U.K. and the other unspecified.

**Figure 39:**
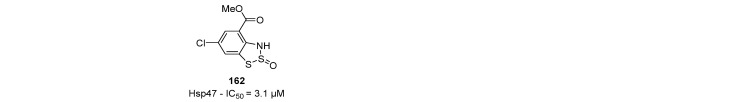
Hsp47 inhibitor methyl 6-chloro-3*H*-benzo[*d*][1,2,3]dithiazole-4-carboxylate 2-oxide (**162**).

In 2005, Ananthanarayanan et al. screened a Maybridge compound library against Heat shock protein 47 (Hsp47), which, at the time, had no known inhibitors. Hsp47 is a collagen-specific molecular chaperone whose activity has been implicated in the pathogenesis of fibrotic diseases. The regulation of both Hsp47 and collagen expression has been implicated in several different disease indications where changes in the collagen expression are found. These diseases include fibrotic diseases of the liver [78], kidney [79], lung [80], and skin [81], in addition to atherosclerosis [82] and cancer [83]. The screen resulted in a primary hit rate of 0.2%, with 4 out of 2080 compounds being shown to be inhibitors of Hsp47. Secondary screening confirmed **162** (Figure 39), as the most potent compound (IC_50_ = 3.1 μM).

#### 3.4.3. Arabidopsis Gibberellin 2-Oxidase Inhibitors

**Figure 40:**
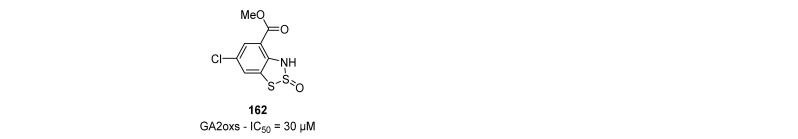
GA2oxs inhibitor methyl 6-chloro-3*H*-benzo[*d*][1,2,3]dithiazole-4-carboxylate 2-oxide (**162**).

In 2010, screening a commercial library of starting points against to Arabidopsis gibberellin 2-oxidases identified compound **162** (Figure 40) [20]. The screening aimed to identify an inhibitor that could both promote Arabidopsis seed germination and seedling growth. Compound **162** was able to do both, without having broad spectrum activity similar to Prohexadione (PHX), which is a broad-spectrum inhibitor of all three 2-oxoglutarate dependent dioxygenase’s (2ODD) that were involved in Gibberellin (GA) production (GA 2-oxidase (GA2oxs), GA 3-oxidase (GA3oxs), and GA20-oxidase (GA20oxs)) [84,85]. The 1,2,3-dithiazole **162** was shown to have inhibition GA2oxs with a high degree of specificity, but not on other 2ODDs. The selective inhibition of GA2oxs activity could potentially lead to the delay of GA catabolism in plants, and hence, extend the life of endogenous GA.

## 4. Summary and Overview

The initial observation of the 1,2,3-dithiazole salt 4,5-dichloro-1,2,3-dithiazolium chloride in 1957 [1], was followed by detailed characterization in 1985 [4], and came to be known as Appel salt (**1**) post-1990s [5]. Appel salt (**1**) allowed for one-step access to a range of different chemistries to furnish a wide scope of 5-substituted-1,2,3-dithiazole derivatives [4,5,9,15,27,30,31,32,33,34,35,36,37]. While several additional methods also exist to access C4 substituted derivatives, the main screening has been done on 4-chloro derivatives until more recently [6,12,13,14,40]. However, synthetic challenges remain, including expanding the chemical space including effective synthesis of *N*-alkyl-5*H*-1,2,3-dithiazol-5-imine analogues, and effective access to 4-pyridyl analogues in good yields [37].

The first screening was carried out by Chevron Research Co. in 1977 [2,3], this relatively detailed study has been the foundation of the phenotypic biology observed on this scaffold. It described detailed work on a series of herbicidal effects and anti-mite efficacy, in addition to antifungal activities. This work was followed up in the late 1990s and 2000s by a series of groups extending the understanding of the antifungal and antibacterial SAR scope of the 1,2,3-dithiazole scaffold [15,50,51,52,53]. Interestingly, a patent in 1997 by Rohm & Haas Co. [51] highlighted a potential coating application for the 1,2,3-dithiazole with the discovery of potent antialgae and Gram-negative bacteria inhibition. In 2012, a patent filed by Bayer Cropscience AG presented a much broader library of heteroaromatic derivatives [54], highlighting a wider range of antifungal activities, with high degrees of control of commercially important fungi for crop protection. More recently, the antifungal and antibacterial screening has focused on clinically relevant hospital derived infections with good efficacy [86], in part aided by a series of matched pair 1,2,3-thiaselenazoles [55].

More recently, several other phenotypic observations have been reported. These include antiviral efficacy against FIV as a model for HIV, where modelling and mechanistic rationale point to cystine containing nucleocapsid protein (NCp) as the target for the 1,2,3-dithiazole [17,36,63]. Anticancer effects against a broad range of cancer cell lines have also been reported with limited off-target toxicity [13,16,74]. These were also supported by modelling and mechanistic rationale highlighting ASCT2 [65] and LAT1 [70] as potential targets responsible. This rationale has been further supported by a series of mechanism of action experiments [65,70,73].

In addition to these reports, a series of other studies also highlighted other activities of the 1,2,3-dithiazole scaffold. These included an anti-melanin phenotype in *Xenopus laevis* embryos, where active potent (>5 μM) non-toxic compounds were identified in an in vivo model [19]. An ANRONC style mechanism of action was proposed supported by a series of chemical modifications to the scaffold [38,39,40]. Finally, two reports of high-throughput screens identified hit compounds against antifibrotic collagen specific chaperone hsp47 [18] and Arabidopsis Gibberellin 2-Oxidase [20].

**Figure 41:**
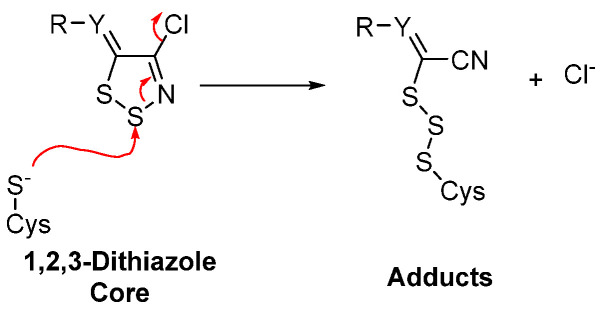
The 1,2,3-dithiazole as a latent cystine reactive functionality.

The full potential of the 1,2,3-dithiazole scaffold has yet to be realized. Key areas of biological activities have been identified with preliminary work in the literature showing encouraging results. These included activities as antifungal [2], herbicidal [2], antibacterial [15], anticancer [16], antiviral [17], antifibrotic [18], and being a melanin [19] and Arabidopsis gibberellin 2-oxidases [20] inhibitors. These results provide a prospective to the versatility as to what is possible with this scaffold. In addition to these interesting reported biology applications, there are potentially significant untapped chemical biology opportunities towards targeting cystine reactive sites [21,22,23,24,25]; using the ANRORC-style 1,2,3-dithiazole chemistry as a latent functionality (Figure 41).

## 5. Conclusions

Taken together, the chemistry and biology of the 1,2,3-dithiazoles chemotype has shown a lot of exciting potential. The ANRORC-style rearrangements potentially affording a new route for potential chemical tools and relative cystine within proteins pockets, while the sub-micro molar phenotypic potencies against a series of diverse targets demonstrate potential for further development. Many of these diseases and pathogens have limited treatment options and need new therapies with novel mechanisms of action. The identification of starting points and defined SAR provides the foundation to define a medicinal chemistry trajectory towards optimized inhibitors and potential new treatments for a broad range of diseases.

## Data Availability

Not applicable.
